# Heme oxygenase-2 (HO-2) binds and buffers labile ferric heme in human embryonic kidney cells

**DOI:** 10.1016/j.jbc.2021.101549

**Published:** 2021-12-29

**Authors:** David A. Hanna, Courtney M. Moore, Liu Liu, Xiaojing Yuan, Iramofu M. Dominic, Angela S. Fleischhacker, Iqbal Hamza, Stephen W. Ragsdale, Amit R. Reddi

**Affiliations:** 1 School of Chemistry and Biochemistry, Georgia Institute of Technology, Atlanta, Georgia, USA; 2 Department of Biological Chemistry, University of Michigan, Ann Arbor, Michigan, USA; 3 Department of Animal and Avian Sciences, Department of Cell Biology and Molecular Genetics, University of Maryland, College Park, Maryland, USA; 4 School of Biological Sciences, Georgia Institute of Technology, Atlanta, Georgia, USA; 5 Parker Petit Institute for Bioengineering and Biosciences, Georgia Institute of Technology, Atlanta, Georgia, USA

**Keywords:** heme homeostasis, heme oxygenases, heme oxygenase-2, heme trafficking, labile heme, ALA, 5-aminolevulinic acid, CO, carbon monoxide, CPR, cytochrome P450 reductase, DMSO, dimethylsulfoxide, DPBS, Dulbecco’s Phosphate Buffered Saline, eGFP, enhanced green fluorescent protein, ER, endoplasmic reticulum, FBS, fetal bovine serum, HD, heme deficient, HO, heme oxygenase, HRMs, heme regulatory motifs, HS1, heme sensor 1, LH, labile heme, SA, succinylacetone, siRNA, small interfering RNA

## Abstract

Heme oxygenases (HOs) detoxify heme by oxidatively degrading it into carbon monoxide, iron, and biliverdin, which is reduced to bilirubin and excreted. Humans express two isoforms of HO: the inducible HO-1, which is upregulated in response to excess heme and other stressors, and the constitutive HO-2. Much is known about the regulation and physiological function of HO-1, whereas comparatively little is known about the role of HO-2 in regulating heme homeostasis. The biochemical necessity for expressing constitutive HO-2 is dependent on whether heme is sufficiently abundant and accessible as a substrate under conditions in which HO-1 is not induced. By measuring labile heme, total heme, and bilirubin in human embryonic kidney HEK293 cells with silenced or overexpressed HO-2, as well as various HO-2 mutant alleles, we found that endogenous heme is too limiting a substrate to observe HO-2-dependent heme degradation. Rather, we discovered a novel role for HO-2 in the binding and buffering of heme. Taken together, in the absence of excess heme, we propose that HO-2 regulates heme homeostasis by acting as a heme buffering factor that controls heme bioavailability. When heme is in excess, HO-1 is induced, and both HO-2 and HO-1 can provide protection from heme toxicity via enzymatic degradation. Our results explain why catalytically inactive mutants of HO-2 are cytoprotective against oxidative stress. Moreover, the change in bioavailable heme due to HO-2 overexpression, which selectively binds ferric over ferrous heme, is consistent with labile heme being oxidized, thereby providing new insights into heme trafficking and signaling.

Heme is an essential but potentially cytotoxic metallocofactor and signaling molecule (1, 2, 3, 4, 5, 6, 7, 8, 9, 10). Consequently, cells must tightly regulate the concentration and bioavailability of heme (8, 11, 12, 13, 14). In mammals, the total intracellular concentration of heme is governed by the relative rates of *de novo* synthesis, degradation, import, and export. The atomic resolution structures and chemical mechanisms of all the heme biosynthetic and catabolic enzymes are known and well understood (11, 15). Although cell surface heme importers (16) and exporters (17, 18) have been identified, their molecular mechanisms remain poorly characterized and outside of developing red blood cells in the case of heme exporters, the physiological context in which they function is unclear and controversial (19). The bioavailability of heme, which is comparatively less well understood, is governed by a poorly characterized network of heme buffering factors, intracellular transporters, and chaperones that ensure heme is made available for heme-dependent processes located throughout the cell.

When the cells are confronted with excess heme, heme synthesis is downregulated (11, 20, 21), and heme can be detoxified by storage into lysosome-related organelles (22, 23), export (24, 25), or degradation (26, 27, 28, 29, 30, 31). Arguably, the best understood mechanism for heme detoxification is through the heme catabolism pathway. The first and rate-limiting step of heme degradation is catalyzed by the heme oxygenases (HO) (32, 33). Mammals encode two HO isoforms, inducible HO-1 and constitutive HO-2 (34, 35, 36, 37). HO-1 and HO-2 are structurally similar, both in primary sequence and tertiary structure, operate using the same chemical mechanism, and exhibit similar catalytic properties, including Michaelis constants (*K*_M_) and maximal velocities (*V*_max_) (37, 38, 39). HOs, which are primarily anchored into the endoplasmic reticulum (ER) membrane and whose active sites face the cytoplasm, bind oxidized ferric heme in its resting state using a histidine axial ligand. Upon reduction, using electrons from the NADPH-cytochrome P450 reductase (CPR) system and dioxygen binding (O_2_), HOs catalyze the oxidative degradation of heme to form biliverdin, ferrous iron (Fe^2+^), and carbon monoxide (CO) (40, 41, 42, 43, 44). Biliverdin is subsequently rapidly metabolized to bilirubin *via* a NADPH-biliverdin reductase and expelled from the cells (45, 46). Given that heme catabolites ferrous iron, CO, biliverdin, and bilirubin have their own distinct beneficial or detrimental effects on cell physiology in various contexts, the activity of HO enzymes and availability of its heme substrate can impact metabolism in numerous ways (32, 47, 48, 49, 50, 51, 52, 53, 54, 55).

Although the structures and mechanisms of HO-1 and HO-2 are largely the same (37, 38, 39, 41, 43, 56), the regulation and expression of these two enzymes is very different (27, 31, 35). Heme oxygenase-1, which is comparatively far better understood, is induced by excess heme, as well as several nonheme stressors like oxidative stress, infection, and exposure to various xenobiotics (57, 58, 59, 60, 61). HO-2, on the other hand, is constitutively expressed across all tissues and cell types, being most abundant in the brain and testis (34, 35). The current rationale for dual mammalian HO isoforms is that HO-2 provides a baseline level of protection from heme in the absence of cellular stressors that would otherwise induce HO-1. However, the biochemical necessity for expressing constitutive HO-2 is largely dependent on whether sufficient heme is available as a substrate under conditions in which HO-1 is not induced.

Total cellular heme in yeast and various nonerythroid human cell lines is on the order of 1 to 20 μM (62, 63, 64, 65, 66, 67). All heme in the cell partitions between exchange inert high affinity hemoproteins, such as cytochromes and other heme enzymes, and certain exchange labile heme (LH) complexes that buffer free heme down to nanomolar concentrations (8, 12, 13, 14, 63, 68, 69). The factors that buffer heme are poorly understood, but likely consist of a network of heme-binding proteins, nucleic acids, and lipid membranes (8, 12, 13, 14, 63, 66, 67, 70, 71). Labile heme may act as a reservoir for bioavailable heme that can readily exchange with and populate heme-binding sites in heme dependent or regulated enzymes and proteins. The nature of LH, including its speciation, oxidation state, concentration, and distribution are not well understood but may be relevant for the mobilization and trafficking of heme. It is currently not known what the source of heme is for HOs, that is, whether it is buffered-free heme or a dedicated chaperone system that traffics and channels heme to HO in a manner that bypasses the LH pool.

The recent development of fluorescence and activity-based heme sensors has offered unprecedented insights into LH and their diverse roles in physiology (63, 68, 69, 72, 73). Strictly speaking, these probes report on the *availability* of heme to the sensor, not necessarily *free* heme coordinated by water (8, 13, 74). In other words, the heme occupancy of the sensor is dictated by the extent to which LH can exchange with the probe. However, many investigators convert the fractional heme loading of a probe to a buffered-free heme concentration, which can be done if the heme-sensor dissociation constant is known. Although problematic in that the sensor may not be probing “free heme”, it nonetheless provides a measure of labile or accessible heme because the calculated concentration of free heme is related to sensor heme occupancy.

In intact living yeast and various nonerythroid human cell lines, the estimates of buffered-free heme based on genetically encoded heme sensors are on the order of ∼5 to 20 nM (63, 68, 69). If free heme is a heme source for HO-2, which has a *K*_M_ value of 400 nM for heme (37), it is expected to be less than 5% active (Fig. 1, black curve), assuming other HO substrates, for example, CPR and O_2_ are not limiting. In contrast, using heme reporters in human embryonic kidney HEK293 and human lung fibroblast IMR90 cell extracts, it was found that buffered-free heme was as high as 400 to 600 nM (73, 75), corresponding to heme concentrations in which HO-2 is ∼50% active (Fig. 1, black curve). Given the relatively large span in estimates of free heme using different detection methods, between 20 and 600 nM, it is not clear how active HO-2 is, raising the intriguing possibility that it may have alternative roles in heme cell biology distinct from enzymatic heme degradation. Indeed, prior reports have found that enzymatically inactive HO-2 alleles can rescue cells from oxidative stress (76). Alternatively, if heme were delivered to HO-2 using a specific heme delivery system that bypasses LH, the activity of HO-2 would be dependent on access to such heme chaperones, making LH or buffered-free heme irrelevant toward understanding the activity of HO-2 in cells.

**Figure 1:**
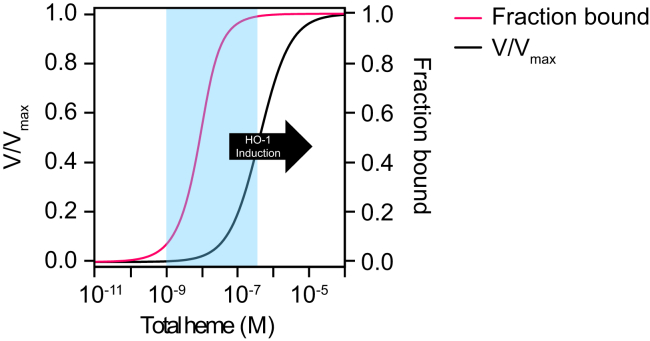
**Fractional heme occupancy and activity of HO-2 as a function of heme concentration.** The heme occupancy of HO-2 (*pink curve*; *right* y-axis) was simulated using the 1-site heme-binding model (111, 112): $\frac{\left[\text{HO}-2-\text{Heme}\right]}{{\left[\text{HO}-2\right]}_{\text{Total}}}=\frac{\left\{{\left[\text{HO}-2\right]}_{\text{Total}}+{\left[\text{Heme}\right]}_{\text{Total}}+K_{\text{D}}\right\}\;-\;\sqrt{\left\{{\left[\text{HO}-2\right]}_{\text{Total}}+{\left[\text{Heme}\right]}_{\text{Total}}+K_{\text{D}}\right\}{\;}^{2}-4{\left[\text{HO}-2\right]}_{\text{Total}}{\left[\text{Heme}\right]}_{\text{Total}}}}{2{\left[\text{HO}-2\right]}_{\text{Total}}}$. The fraction of maximal velocity, V/V_max_, (*black curve*; *left* y-axis) was simulated using the quadratic velocity equation for tight binding substrates (113): $\frac{\left[\text{V}\right]}{{\left[\text{V}\right]}_{\text{Max}}}=\frac{\left\{{\left[\text{HO}-2\right]}_{\text{Total}}+{\left[\text{Heme}\right]}_{\text{Total}}+K_{\text{M}}\right\}\;-\;\sqrt{\left\{{\left[\text{HO}-2\right]}_{\text{Total}}+{\left[\text{Heme}\right]}_{\text{Total}}+K_{\text{M}}\right\}{\;}^{2}-4{\left[\text{HO}-2\right]}_{\text{Total}}{\left[\text{Heme}\right]}_{\text{Total}}}}{2{\left[\text{HO}-2\right]}_{\text{Total}}}$. These simulations were parameterized using the previously determined heme-HO-2 *K*_D_ and *K*_M_ value of 3.6 nM (77) and 400 nM (37), respectively, and [HO-2]_Total_ = 10 nM, which was determined in the present study for HEK293 cells (*vida infra*). These models both assume the following mass balance: [Heme]_Total_ = [HO-2-Heme] + [Heme] and [HO-2]_Total_ = [HO-2-Heme] + [HO-2]. Moreover, the quadratic velocity equation assumes that the other HO-2 substrates, CPR and O_2_, are not limiting, and that the HO-2-Heme Michaelis complex is generated as quickly as it is consumed, that is, the steady-state approximation. The *light blue shaded region* represents the span of values reported for buffered free heme in cells and the *black arrow* indicates the concentration of intracellular free heme in which HO-1 is induced. CPR, cytochrome P450 reductase; HO, heme oxygenase.

An alternative model for constitutive HO-2 function is that it acts as a component of a larger network of proteins that buffer heme. The ferric heme dissociation constant (*K*_D_^III^) for HO-2 is 3.6 nM (77) and, at the lower estimates of buffered-free heme concentrations, is expected to be fractionally populated with heme (Fig. 1, pink curve). As such, HO-2 may act within a network of LH complexes and serve as an access point for heme distribution, possibly to the ER. Such a model would require that LH is largely oxidized and exchangeable with HO-2 and would predict that perturbations in HO-2 expression will alter LH or buffered-free heme.

In the present report, using the model human cell line, HEK293 cells, we sought to determine if endogenous LH is sufficiently accessible and abundant for changes in HO-2 expression to impact heme degradation and establish the oxidation state of LH. HEK293 cells were chosen because of extensive prior work in these cell lines probing HO-2 function (64, 76, 78) and LH (67, 68, 69, 73). By measuring LH, total heme, and bilirubin in HEK293 cells with silenced or over-expressed HO-2, and various mutant HO-2 alleles, we found that heme is accessible to HO-2, but too limiting to observe HO-2-dependent alterations in heme degradation. Rather, our data support a role for HO-2 in regulating heme bioavailability by buffering it. Moreover, the change in LH due to HO-2 overexpression is consistent with LH being largely oxidized. Altogether, our findings force us to rethink the physiological role of constitutive HO-2 in cell types that have buffered-free heme levels well below its heme *K*_M_ values.

## Results

### Measuring LH in HEK293 cells

To determine how active HO-2 is in HEK293 cells, we first sought to determine the amount of cytosolic LH. Toward this end, we transiently transfected previously described genetically encoded fluorescent heme sensors into HEK293 cells and analyzed LH levels using flow cytometry. Heme sensor 1 (HS1) is a tri-domain construct consisting of a heme-binding domain, cytochrome *b*_562_ (Cyt *b*_562_), an enhanced green fluorescent protein (eGFP) whose emission is quenched by heme, and a red fluorescent protein (mKATE2) whose emission is relatively unaffected by heme (63). Thus, the eGFP/mKATE2 fluorescence ratio of HS1 decreases upon heme binding and is a reporter of the buffered exchange labile or bioavailable heme pool in cells.

**Figure 2:**
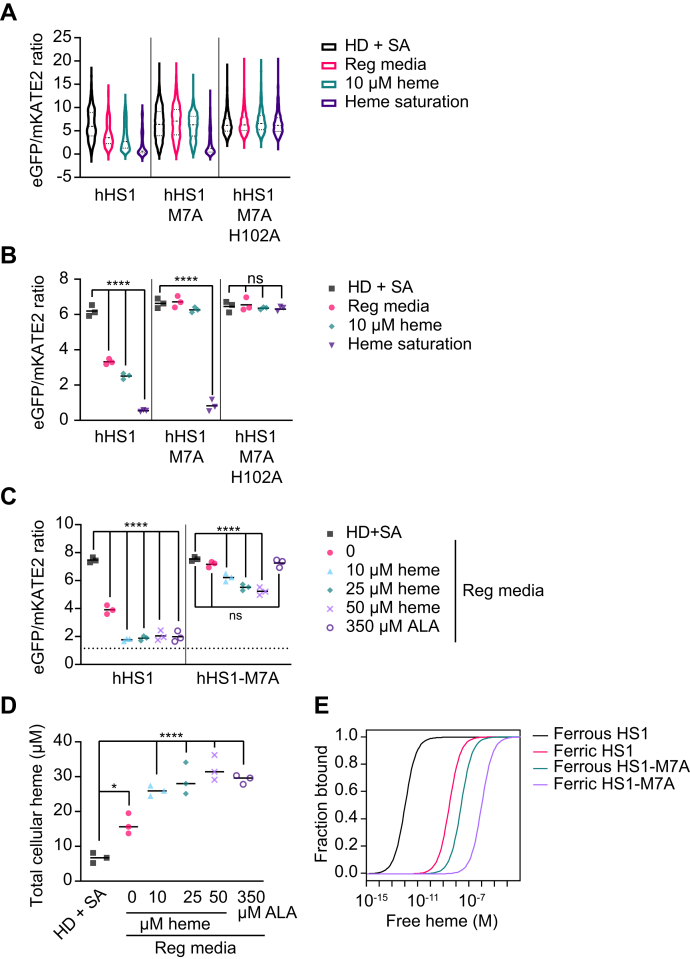
**Heme sensor 1 (HS1) can sense labile heme in HEK293 cells.***A*, representative violin plots depicting the distribution of eGFP/mKATE2 fluorescence ratios in single HEK293 cells expressing the high affinity heme sensor, HS1, the moderate affinity heme sensor, HS1-M7A, and the heme-binding incompetent control scaffold, HS1-M7A, H102A. The heme responsiveness of the sensors was established by culturing HEK293 cells in regular media (reg. med.; DMEM with 10% v/v FBS), heme deficient (HD) media supplemented with succinylacetone (SA) (HD + SA; DMEM with heme depleted 10% v/v FBS and 500 μM SA), or in regular media with 10 μM hemin chloride (10 μM heme). To saturate the sensors, the cells were permeabilized with digitonin and incubated with 100 μM hemin chloride (heme saturation; serum-free DMEM with 1 mM ascorbate, 40 μM digitonin, and 100 μM hemin chloride). *B*, median heme sensor eGFP/mKATE2 fluorescence ratio values derived from flow cytometry experiments (as in *A*) from triplicate HEK293 cultures. *C*, median heme sensor eGFP/mKATE2 fluorescence ratio values derived from flow cytometry experiments from triplicate HEK293 cultures grown in HD + SA media or in regular media supplemented with the indicated concentrations of hemin chloride or 5-aminolevulinic acid (ALA) for 24 h. Representative violin plots of eGFP/mKATE2 fluorescence ratio distributions from single cell analysis of HEK293 cultures are shown in Fig. S8*A*. *D*, measurements of total heme in triplicate HEK293 cultures grown in HD + SA media or in regular media supplemented with the indicated concentrations of hemin chloride or ALA for 24 h. *E*, relationship between sensor heme occupancy for HS1 and HS1-M7A and buffered-free heme, depending on weather heme is oxidized (ferric) or reduced (ferrous). See main text and Experimental procedures for details. The statistical significance is indicated by *asterisks* using one-way ANOVA for multiple comparisons using Tukey’s range test. ∗*p* = 0.0140, ∗∗∗∗*p* < 0.0001, ns = not significant. eGFP, enhanced green fluorescent protein; FBS, fetal bovine serum.

We analyzed intracellular LH using the high affinity heme sensor, HS1, which binds heme using Cyt *b*_562_ Met_7_ and His_102_, a moderate affinity heme sensor, HS1-M7A, and a variant that cannot bind heme, HS1-M7A,H102A (63). Between pH 6.8 to 7.4, the consensus range of cytosolic pH values reported for HEK293 cells (79, 80, 81), the ferric and ferrous heme dissociation constants for HS1 are *K*_D_^III^ = 3 nM and *K*_D_^II^ = 1 pM and for HS1-M7A are *K*_D_^III^ = 1 μM and *K*_D_^II^ = 25 nM (62, 63). As shown in Figure 2, *A* and *B*, flow cytometric analysis of HS1 eGFP/mKATE2 fluorescence ratios indicate that it is heme responsive. Compared to cells cultured in regular media (reg media), HS1 expressed in HEK293 cells depleted of intracellular heme by culturing in heme deficient (HD) media and with the heme biosynthetic inhibitor succinylacetone (SA) for 24 h have a characteristically high median eGFP/mKATE2 fluorescence ratio. In contrast, the cells cultured with 10 μM heme for 24 h have a characteristically low median eGFP/mKATE2 fluorescence ratio (Fig. 2, *A* and *B*). On the other hand, HS1-M7A and HS1-M7A,H102A are not heme responsive because eGFP/mKATE2 fluorescence ratios were not sensitive to heme depletion (HD+SA) or heme excess (10 μM heme) (Fig. 2, *A* and *B*). *In toto*, assessment of the median eGFP/mKATE2 fluorescence ratios from replicate flow cytometry data (Fig. 2*B*) indicate that HS1 binds heme tightly enough to measure endogenous LH in cells, but HS1-M7A does not.

Owing to the relatively broad, non-normal distribution of heme sensor ratios within a population of cells, the median sensor ratio values are reported, which are not as sensitive to outliers as the mean. Consequently, the analysis between experimental groups involves comparing the average median sensor ratios from replicates. The relatively broad distribution in sensor ratio is expected given the heterogeneity in subcellular heme between cells within a population. For instance, prior studies with stably integrated heme sensors in HeLa-C9C cells (68) and low copy expression plasmids in *Saccharomyces cerevisiae* (63) also revealed a broad distribution in sensor ratio and heme levels. In addition, cell-to-cell variability in microenvironment may cause alterations in fluorescence (82, 83, 84), giving rise to heme-independent variations in sensor ratio. Indeed, such differences in eGFP and mKATE2 fluorescence, independent of heme binding, likely accounts for the distribution in fluorescence ratios observed for HS1-M7A,H102A, and in-part, the spread in HS1 and HS1-M7A sensor ratios.

To better quantify LH, we adapted a previously developed sensor calibration protocol to relate the sensor fluorescence ratios to its heme occupancy (63). The fractional saturation of the heme sensor is governed by Equation 1 (63):

(1) $$\%\text{ Heme Occupancy}=\frac{R-R_{\text{min}}}{R_{\text{max}}-R_{\text{min}}}\times 100$$

where R is the eGFP/mKATE2 fluorescence ratio under any given experimental condition, *R*_max_ is the eGFP/mKATE2 fluorescence ratio when the sensor is saturated with heme, and *R*_min_ is the eGFP/mKATE2 fluorescence ratio when the sensor is depleted of heme. *R*_max_ is determined by permeabilizing cells with digitonin and adding an excess of heme to drive heme binding to the sensor (“Heme Saturation” in Fig. 2, *A* and *B*). *R*_min_ is determined by growing cells with SA in media that was depleted of heme (HD) (“HD + SA” in Fig. 2, *A* and *B*). Analysis of the median fluorescence ratios from replicate flow cytometry measurements (Fig. 2*B*) indicates that the heme occupancies of HS1 and HS1-M7A are ∼50% and ∼0%, respectively, when HEK293 cells are cultured in regular media.

HS1 can be saturated to near 100% heme occupancy in cells supplemented with 350 μM 5-aminolevulinic acid (ALA), a heme biosynthetic precursor that induces heme synthesis, or with 10 to 50 μM exogenous heme (Fig. 2*C*), both of which lead to accumulation of up to 30 μM intracellular heme (Fig. 2*D*). Consequently, ALA supplementation can also be used as a calibration control to saturate HS1 in control experiments to establish % heme occupancy. In contrast, HS1-M7A heme occupancy remains at ∼0% with ALA supplementation and only increases to ∼30% fractional heme saturation when intracellular heme approaches 30 μM with exogenous heme supplementation (Fig. 2, *C* and *D*).

If one assumes the sensor is binding *free* heme, the concentration of buffered free heme can be determined by Equation 2 (63):

(2) $$\left[\text{Heme}\right]=\;\frac{\;\alpha \;\times \;K_{D}}{100-\alpha }$$

where *K*_D_ is the heme sensor–heme dissociation constant and α is the heme occupancy of the sensor defined by Equation 1. If one assumes LH is largely reduced, the buffered-free heme concentration can be estimated to be ∼1 pM in HEK293 cells cultured in regular media by assuming the HS1 median *R*_min_, *R*_max_, and R values from Figure 2*B* or Figure 2*C*, as well as the HS1 *K*_D_^II^ value of 1 pM. On the other hand, if one assumes LH is largely oxidized, the buffered-free heme concentration can be estimated to be ∼5 nM, assuming a HS1 *K*_D_^II^ value of 3 nM and the aforementioned median *R*_min_, *R*_max_, and R values from Figure 2*B* or Figure 2*C*.

Uncertainties in the concentration of free heme stem from the broad distribution in heme sensor ratios, a lack of knowledge of the specific oxidation state of LH, which is addressed further below, and a means to precisely determine *R*_min_. The variation in the concentrations of free heme can be estimated based on the HS1 and HS1-M7A heme occupancies, their ferric or ferrous heme dissociation constant values, and assumptions about the oxidation state of LH (Fig. 2*E*). Regarding *R*_min_, because heme is absolutely required for human cell lines, heme depletion by culturing cells in HD+SA media does not completely eliminate intracellular heme and results in only a 2-fold decrease compared to cells cultured in regular media (Fig. 2*D*) (64). Past studies in *S. cerevisiae*, which can be cultured in the complete absence of heme if supplemented with oleic acid and ergosterol, found that LH is depleted by > 90% when total heme is diminished by only 2-fold due to SA treatment (62). The degree to which HEK293 cells are yeast-like in the sensitivity of their LH pools to heme depletion will dictate the accuracy of *R*_min_ and therefore sensor heme occupancy and [LH]. Irrespective of the precise values of buffered-free heme and *R*_min_, the eGFP/mKATE2 fluorescence ratios of HS1, relative to *observed R*_min_ and *R*_max_ values, can nonetheless be used as a readout of sensor heme loading and exchange LH.

One additional concern is the possibility that the expression of the heme sensor will itself perturb heme homeostasis by removing heme from other hemoproteins and heme-binding sites. The extent to which the heme reporter can impact free or LH is related to the sensor expression level and heme affinity, the degree to which heme can equilibrate between the sensor and competing proteins and the concentration and relative heme affinities of competing hemoproteins. If the heme sensor is limiting and is below the concentration of the total heme being sensed, the heme reporter will not significantly impact *apparent* heme availability, including LH and free heme. This competition equilibria is modeled in Fig. S1, assuming total heme is oxidized and has a concentration of 15 μM, which is similar to total heme levels found in yeast and various nonerythroid human cell lines (63, 64, 67), including HEK293 cells (this study), a generic heme buffer (B) having a heme *K*_D_ value of 10 nM, and a heme sensor *K*_D_ value of 3 nM (62, 63). Simulations were run using ChemEQL (v. 3.2.1) (85, 86) and conducted for concentrations of heme buffer “B” spanning 10-, 5-, and 1-times the total heme concentration. The simulations reveal that for heme sensor concentrations below 1 μM, there is a negligible effect on free heme and LH. Given that HS1 is expressed at levels spanning 10 to 100 nM (63, 67), we do not expect that HS1 significantly perturbs heme homeostasis. Indeed, past studies found that HS1 expression does not affect cell viability and proliferation or perturb the activities of heme-dependent enzymes and pathways, including catalase activity, respiration, and total heme levels, thereby suggesting the sensor does not affect heme-related metabolism (63). However, it is important to note that the model assumes that the total amount of heme in cells can equilibrate with the heme buffer and sensor, which (very likely) may not be the case due to compartmentalization and partitioning of heme between exchange inert and labile-binding sites. Moreover, the identity of the constituent components of the heme buffer, that is, LH complexes, and their heme affinities are unknown. Thus, the true extent to which the heme reporter could impact heme homeostasis is unclear given the uncertainty in heme speciation.

### Heme oxygenase-2 regulates heme bioavailability but not heme degradation in HEK293 cells

**Figure 3:**
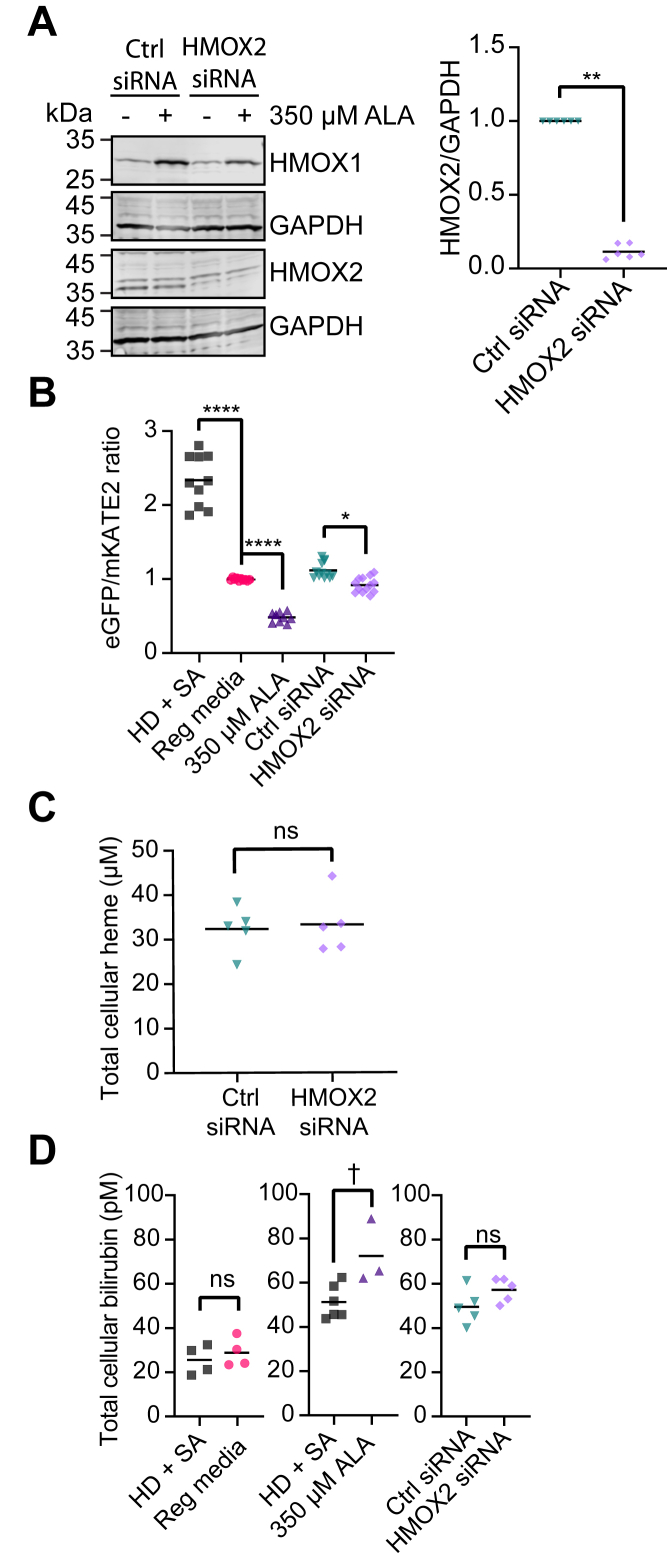
**HO-2 silencing increases labile heme but does not affect total heme or bilirubin levels.***A*, representative immunoblot of HO-1 (HMOX1), HO-2 (HMOX2), and GAPDH expression in HEK293 cells treated with or without 350 μM 5-aminolevulinic acid (ALA) and scrambled (Ctrl siRNA) or targeted (HMOX2 siRNA) siRNA against HO-2 (*left*). Immunoblot analysis from six independent trials demonstrates that siRNA against HMOX2 results in ∼80 to 90% silencing of HO-2 protein expression (*right*). *B*, median heme sensor eGFP/mKATE2 fluorescence ratio values derived from flow cytometry experiments from 9 to 13 replicates of HEK293 cultures grown in HD + SA media, regular media, or regular media supplemented with 350 μM ALA or control or targeted siRNA against HMOX2. Representative violin plots of eGFP/mKATE2 fluorescence ratio distributions from single cell analysis of HEK293 cultures are shown in Fig. S8*B*. *C*, measurements of total heme in quintuplicate HEK293 cultures grown in regular media supplemented with control or targeted siRNA against HMOX2. *D*, measurements of total bilirubin in 3 to 6 replicates of HEK293 cultures grown in in HD + SA media, regular media, or regular media supplemented with 350 μM ALA or control or targeted siRNA against HMOX2. The statistical significance is indicated by *asterisks* using one-way ANOVA for multiple comparisons using Tukey’s range test. ∗*p* = 0.0376, ∗∗*p* = 0.0022, ^†^*p* = 0.0156, ∗∗∗∗*p* < 0.0001, ns = not significant. eGFP, enhanced green fluorescent protein; HD, heme deficient; HO, heme oxygenase; SA, succinylacetone.

Having established that we can probe LH in HEK293 cells using HS1, we next sought to determine the effects of HO-2 silencing on the intracellular concentrations of free heme, total heme, and bilirubin, a readout of HO activity. Silencing HO-2 using small interfering RNA (siRNA) resulted in ∼80% depletion of steady-state HO-2 levels and did not alter HO-1 expression (Fig. 3*A*). Heme oxygenase-2 silencing results in a small but statistically significant increase in LH, with the median eGFP/mKATE2 fluorescence ratio decreasing from 1.1 to 0.9, which corresponds to an 11% increase in HS1 heme occupancy, from 63% to 74% (Fig. 3*B*). In contrast, the cellular heme-degrading activity of HOs are not affected by HO-2 as evidenced by total heme (Fig. 3*C*) and bilirubin levels (Fig. 3*D*) being unchanged by ablating HO-2. Heme-degrading activity only increases when the cells are challenged with elevated intracellular heme. Cells cultured with 350 μM ALA, which increases total and LH (Fig. 2*D*), exhibit significantly elevated bilirubin levels (Fig. 3*D*). Cells cultured with ALA have elevated HO-1 expression, up to ∼10-fold, with no change in HO-2 levels (Fig. 3*A*). In total, our data indicate that HO-2 expression affects LH but not heme degradation in HEK293 cells.

### Heme oxygenase-2 overexpression decreases heme availability in a manner that does not require its catalytic activity

**Figure 4:**
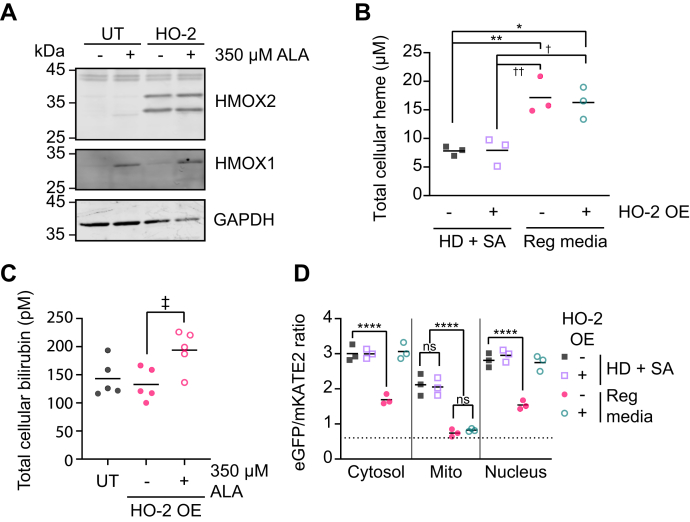
**Heme oxygenase-2 overexpression depletes cytosolic and nuclear labile heme but not total heme, bilirubin levels, or mitochondrial labile heme.***A*, representative immunoblot of HO-1 (HMOX1), HO-2 (HMOX2), and GAPDH expression in untransfected (UT) or HO-2 overexpressing HEK293 cells treated with or without 350 μM ALA. *B*, measurements of total heme in triplicate untransfected (-) or HO-2 overexpressing (OE) (+) HEK293 cultures grown in HD + SA or regular media. *C*, measurements of total bilirubin in replicate untransfected (-) or HO-2 overexpressing (OE) (+) HEK293 cultures grown in regular media with or without 350 μM ALA. *D*, median cytosolic, nuclear, or mitochondrial (mito)-targeted HS1 eGFP/mKATE2 fluorescence ratio values derived from flow cytometry experiments from triplicate untransfected (-) or HO-2 overexpressing (OE) (+) HEK293 cells grown in HD + SA or regular media. Representative violin plots of eGFP/mKATE2 fluorescence ratio distributions from single cell analysis of HEK293 cultures are shown in Fig. S8*C*. The statistical significance is indicated by *asterisks* using one-way ANOVA for multiple comparisons using Tukey’s range test. ∗*p* = 0.0136, ∗∗*p* = 0.0078, ^†^*p* = 0.0145, ^††^*p* = 0.0083, ^‡^*p* = 0.0292, ∗∗∗∗*p* < 0.0001, ns = not significant. ALA, 5-aminolevulinic acid; eGFP, enhanced green fluorescent protein; HD, heme deficient; HO, heme oxygenase; HS1, heme sensor 1; SA, succinylacetone.

Having established that HO-2 depletion increases LH, we sought to determine if HO-2 overexpression could decrease it. Toward this end, an HO-2 overexpression plasmid was transiently transfected into HEK293 cells, resulting in greater than 10-fold increase in HO-2 expression, without affecting HO-1 expression (Fig. 4*A*). Overexpression of HO-2 results in the observation of two proteoforms, which were previously ascribed to being full-length HO-2 (higher molecular weight band) and an N-terminal truncated species of HO-2 (lower molecular weight band) (64, 87, 88, 89). Moreover, ALA-induced overproduction of heme does not alter steady-state HO-2 levels like it does with HO-1, which is induced by heme (Fig. 4*A*). Heme oxygenase-2 overexpression does not affect total heme in either heme-deplete or heme-replete conditions (Fig. 4*B*) or alter bilirubin levels (Fig. 4*C*). To rule out the possibility that electron delivery to the HO system *via* CPR may limit HO activity (76, 78), we also assessed bilirubin production upon addition of 350 μM ALA to increase heme synthesis and found HO overexpressing cells had a concomitant increase in bilirubin levels (Fig. 4*C*). Interestingly, HO-2 overexpression significantly depletes LH in the cytosol, resulting in a shift in HS1 heme occupancy from ∼55% to 0% (Fig. 4*D*). Using heme sensors targeted to the nucleus and mitochondrial network (Fig. S2), we found that HO-2 overexpression likewise depletes nuclear LH but does not affect mitochondrial matrix LH (Fig. 4*D*).

**Figure 5:**
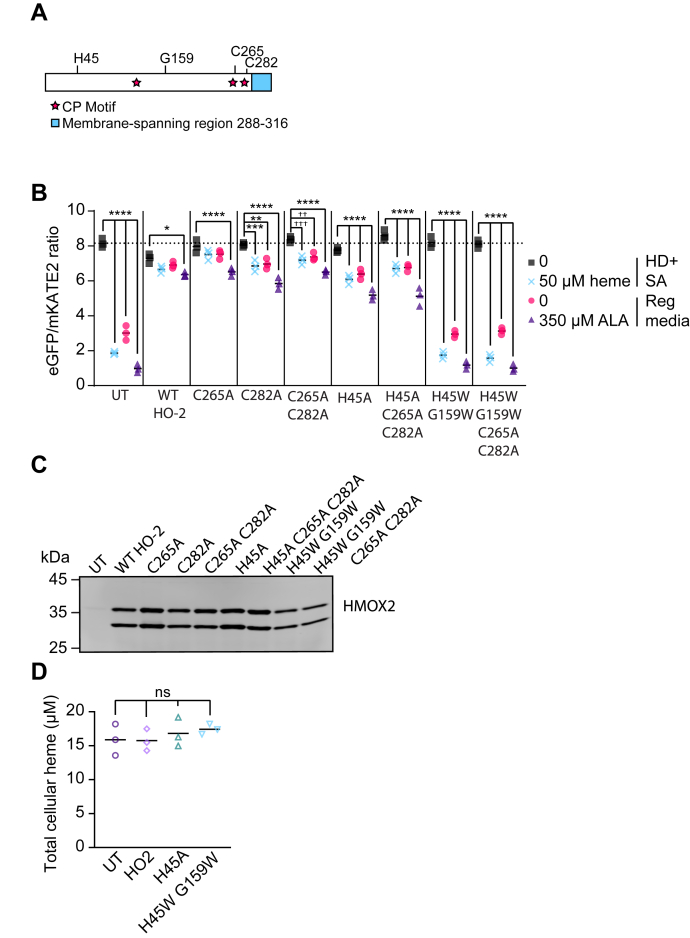
**Heme sequestration and buffering requires the HO-2 heme binding pocket but not catalytic activity or heme regulatory motifs (HRMs).***A*, schematic of HO-2 and residues of interest, including heme-binding ligand H45, a residue that accommodates heme binding within the heme-binding pocket, G159, Cys residues within Cys-Pro (CP; *stars*) dipeptides in the HRMs, C265 and C282, and the ER membrane-spanning region spanning residues 288 to 316 (*blue*). *B*, median cytosolic HS1 eGFP/mKATE2 fluorescence ratio values derived from flow cytometry experiments from triplicate HEK293 cells that were untransfected (UT) or overexpressing WT or the indicated mutant HO-2 alleles. The cells were cultured in HD + SA, treated with or without 50 μM hemin chloride, or regular media, treated with or without 350 μM ALA. Representative violin plots of eGFP/mKATE2 fluorescence ratio distributions from single cell analysis of HEK293 cultures are shown in Fig. S9. *C*, representative immunoblot demonstrating overexpression of the indicated HO-2 (HMOX2) alleles relative to untransfected (UT) cells. *D*, measurements of total heme in triplicate cultures of HEK293 cells that were untransfected (UT) or overexpressing WT or mutant HO-2 grown in regular media. The statistical significance is indicated by *asterisks* using one-way ANOVA for multiple comparisons using Tukey’s range test. ∗*p* = 0.0162, ∗∗*p* = 0.0012, ∗∗∗*p* = 0.0001, ^††^*p* = 0.0068, ^†††^*p* = 0.0003, ∗∗∗∗*p* < 0.0001, ns = not significant. ALA, 5-aminolevulinic acid; eGFP, enhanced green fluorescent protein; ER, endoplasmic reticulum; HD, heme deficient; HO, heme oxygenase; HS1, heme sensor 1; SA, succinylacetone.

We next sought to determine which features of the HO-2 polypeptide are required for its ability to deplete LH when overexpressed (Fig. 5*A*). Mutation of the active site heme-binding histidine residue to alanine (H45A) results in a HO-2 mutant that is catalytically inactive but retains the ability to weakly accommodate heme (64, 90). Overexpression of HO-2 H45A depletes LH, just as WT HO-2 (Fig. 5*B*). However, consistent with the diminution of the HO-2 H45A affinity for heme, there is more LH in cells overexpressing the H45A mutant compared to WT HO-2 in regular media; the HS1 heme occupancies in cells overexpressing WT HO-2 and the H45A mutant are 6% and 20%, respectively.

Heme oxygenase-2 also contains heme regulatory motifs (HRMs), which bind heme through cysteine within a cysteine-proline sequence and often a distal histidine (91, 92). In most proteins, the HRMs regulate protein degradation; however, in HO-2, the HRMs affect protein dynamics (39) and in transfer and loading of heme to the catalytic center (93) but does not otherwise affect protein stability or activity (64). We find that mutation of one or both C-terminal HRMs, C265A and/or C282A, do not affect the ability of overexpressed HO-2 to reduce LH (Fig. 5*B*).

Finally, we tested if mutation of the heme-binding pocket affected LH. Heme oxygenase-2 binds heme within a pocket that provides the aforementioned H45 as a proximal axial ligand for the heme iron center and a distal G159 residue that is hydrogen bonded to an axial water ligand. A H45W/G159W HO-2 mutant, which cannot bind heme due to excessive steric bulk within the heme-binding pocket and is therefore also catalytically inactive (93), is unable to deplete LH when overexpressed (Fig. 5*B*). Importantly, the HO-2 mutations tested still resulted in high levels of steady-state expression of HO-2 (Fig. 5*C*). Only the H45W/G159W mutant exhibited a decrease in protein expression compared to the other HO-2 variants, consistent with a prior study (64). However, the ∼50% decrease in expression of the H45W/G159W mutant is not the reason why this variant cannot deplete LH. If H45W/G159W HO-2 could deplete LH as the other HO-2 variants, but was expressed at half the level, then it would still diminish LH, but to a lower degree relative to the other HO-2 variants. Also of note, localization to the ER (Fig. S3) and total heme levels (Fig. 5*D*) are unaffected by the HO-2 mutations tested (64). Parenthetically, the ER-localization of HO-2 is not required for heme sequestration because a mutant lacking the ER-tethering C-terminal tail still depletes LH (Fig. S4). Altogether, our data indicate that HO-2 overexpression depletes LH due to its ability to bind and sequester heme, not because of its ability to catalytically degrade heme.

### Labile heme is largely oxidized in HEK293 cells

Because HS1, which is ∼50% heme occupied in HEK293 cells, can bind both oxidation states of heme tightly, with *K*_D_^III^ and *K*_D_^II^ values of 3 nM and 1 pM, respectively, the oxidation state of LH was unclear. The corresponding buffered-free heme concentrations would span a range between 1 pM and 5 nM if it were fully reduced or oxidized, respectively (Fig. 2*E*). Because HO-2 selectively binds ferric over ferrous heme, with a *K*_D_^III^ = 3.6 nM (77) and *K*_D_^II^ = 320 nM (94), the observed changes in HS1 heme occupancy upon depletion or overexpression of HO-2 is consistent with LH being largely oxidized. If LH were reduced, changes in HO-2 expression over the concentration span observed in our work would not perturb HS1 heme occupancy due to the large difference in ferrous heme-binding affinities, *K*_D_^HS1^ = 1 pM and *K*_D_^HO-2^ = 320 nM.

**Figure 6:**
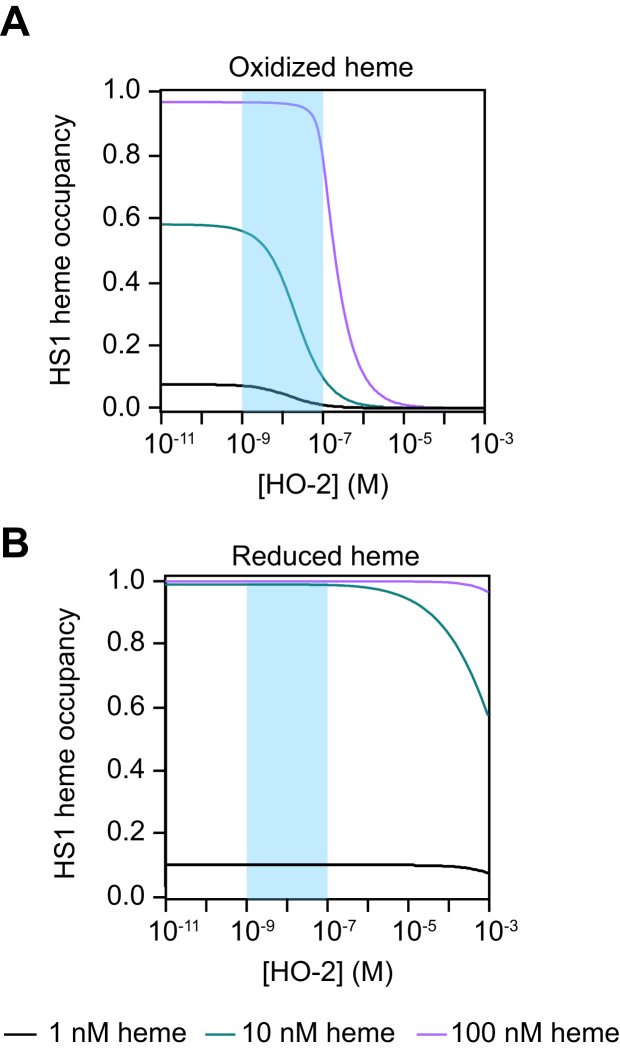
**The effects of HO-2 depletion or overexpression on HS1 heme occupancy are consistent with labile heme being oxidized.***A*, simulation of HS1 heme occupancy as a function of HO-2 expression assuming that heme is oxidized. *B*, simulation of HS1 heme occupancy as a function of HO-2 expression assuming that heme is reduced. Simulations assume the following mass balance terms: [H]_Total_ = [H] + [HO-2-H] + [S-H]; [S]_Total_ = [S] + [S-H]; [HO-2]_Total_ = [HO-2] + [HO-2-H], where H is unbound free heme, S is apo heme sensor, HO-2 represents *apo* HO-2, S-H is the heme bound sensor, and HO-2-H is heme bound HO-2. The heme sensor oxidized and reduced heme K_D_ values were assumed to be 3 nM and 1 pM, respectively. The HO-2-H oxidized and reduced heme K_D_ values were assumed to be 3.6 nM and 320 nM, respectively. The simulations were parameterized by fixing sensor concentration [S]_Total_ = 10 nM, and total heme being present at 1 nM (*black*), 10 nM (*green*), or 100 nM (*purple*). The *blue patch* represents the span in cellular HO-2 concentration accessed upon its silencing (∼1 nM), endogenous expression (∼10 nM), and overexpression (∼100 nM). Simulations were conducted using ChemEQL (v. 3.2.1) (85, 86). HO, heme oxygenase; HS1, heme sensor 1.

To graphically illustrate this point, HS1 heme occupancy as a function of HO-2 expression (Fig. 6) was simulated using ChemEQL (v. 3.2.1) (85, 86) for three concentrations of total heme, [H]_Total_ = 1, 10, or 100 nM. Parameters of the model include the following mass balance terms for heme (H), sensor (S), and HO-2: [H]_Total_ = [H] + [HO-2-H] + [S-H]; [S]_Total_ = [S] + [S-H]; and [HO-2]_Total_ = [HO-2] + [HO-2-H]; ferric heme *K*_D_^HS1^ = 3.0 nM and *K*_D_^HO-2^ = 3.6 nM; and ferrous heme *K*_D_^HS1^ = 1 pM and *K*_D_^HO-2^ = 320 nM. The HO-2 concentration in cells is 10 nM (Fig. S5), with overexpression resulting in a ∼10-fold increase in expression (64) (Fig. 4*A*) and silencing resulting in ∼80% reduction in expression (Fig. 3*A*). If LH was oxidized, alterations in HO-2 expression between 1 to 100 nM would be expected to alter HS1 heme occupancy, as was observed (Fig. 6*A*). On the other hand, if LH were reduced, HO-2 concentrations would have to approach mM levels to alter HS1 heme loading (Fig. 6*B*). In a competition between HS1 and HO-2 for 10 nM total oxidized heme (Fig. 6*A*), the simulation predicts that HS1 heme occupancy increases from 42% to 56% upon silencing of HO-2, which results in a decrease of HO-2 from ∼10 nM to ∼1 nM. This is similar to the 11% increase in HS1 heme occupancy, from 63% to 74%, that was experimentally determined from HO-2 silencing. Deviations in the experimentally observed and simulated changes in HS1 heme occupancy due to HO-2 silencing reflect uncertainty in the speciation of heme, that is, additional competing ligands beyond just HO-2, and the total amount of heme that can equilibrate between HS1, HO-2, and any other LH complexes in the cytosol.

Taken together, the change in HS1 heme occupancy due to HO-2 expression is consistent with LH being oxidized. If one assumed HS1 were sensing free heme, it would translate to a buffered-free ferric heme concentration on the order of 5 nM in HEK293 cells. This conclusion supports our findings that heme may be too limiting to observe HO-2-dependent heme degradation (*K*_M_ = 400 nM) in HEK293 cells and explains why bilirubin and total heme levels are not altered in response to HO-2 depletion or overexpression.

## Discussion

Heme oxygenases are arguably the best characterized systems for degrading and detoxifying excess heme (26, 27, 28, 29, 30, 31). Mammals encode two HO isoforms, an inducible HO-1 and a constitutive HO-2 (32, 33, 34, 35, 36, 37). The current conceptual paradigm for dual HOs is that HO-2 functions to degrade a baseline level of heme under homeostatic conditions, and inducible HO-1 provides additional protection against heme toxicity in response to various stressors, including excess heme, oxidative insults, and exposure to various xenobiotics (57, 58, 59, 60, 61). However, before this work, it was unclear if endogenous heme was sufficiently available as a substrate for HO-2 to support high rates of HO-2 mediated heme degradation (78).

*A priori*, in conceptualizing cellular heme, one can consider total heme to be the sum of the contributions from heme bound to exchange inert and LH complexes and unbound free heme. Therefore, heme availability to HOs is dependent upon its access to certain LH complexes that can exchange with HO or free heme. Degrading heme from exchange inert heme complexes requires a mechanism for heme dissociation, likely involving proteasomal or lysosomal protein degradation and ultimately release of heme into exchange LH complexes or an unbound free state. Given the large complement and concentration of biomolecules that could associate with heme, including proteins, nucleic acids, lipids, and an assortment of small molecules, free heme is buffered to very low concentrations.

The recent development of heme sensors from a number of labs has enabled measurements of chelatable heme. Strictly speaking, these sensors measure exchangeable heme *available* to the sensor, not necessarily *free* heme. Thus, sensor heme occupancy is the best parameter to report when interpreting changes in heme availability. On the other hand, if one assumes cellular chelatable heme is unbound, sensor heme occupancy values can be converted to a buffered free heme concentration. Using a number of different heme reporters across various eukaryotic cell lines, from yeast to a number of nonerythroid human cell lines, buffered-free heme concentrations have been estimated to be between 5 and 600 nM (63, 68, 73, 75). Given this ∼100-fold span in free heme, which is also a proxy for exchange LH, it was not known how active HO-2 would be. Moreover, because HO-2 binds ferric heme in its resting state (41, 42, 43, 44), it was unclear what the oxidation state of the LH pool is or if HO-2 could even equilibrate with it.

Herein, on the basis of the changes in HS1 heme occupancy and the lack of change in total heme and bilirubin levels in response to HO-2 depletion and over-expression in HEK293 cells, we found that LH is largely oxidized and too limiting to observe a prominent role for HO-2 in heme degradation. If assuming HS1 and HO-2 bind free heme, we can estimate a buffered-free heme concentration on the order of 5 nM, which is identical to that reported recently using a peroxidase-based heme reporter in HEK293 cells (69) and makes heme too limiting to observe HO-2 (*K*_M_^Heme^ = 400 nM) dependent heme degradation (37). If assuming HS1 and HO-2 access heme *via* exchange reactions with certain LH complexes, heme exchange with HO-2 is likewise too limiting to observe HO-2-dependent heme degradation. In this case, the HO-2 free heme *K*_M_ value is physiologically irrelevant, and a screen for heme-protein complexes that can exchange with HO-2 and knowledge of their *K*_M_ values with HO-2 is needed to better understand heme substrate availability and HO-2 activity *in vivo*. Regardless of what formalism one uses to contemplate heme availability to HO, that is, free heme *versus* exchangeable heme, we found that HO-2 plays a role in regulating heme bioavailability through its ability to bind but not degrade heme. Our results have a number of important physiological implications for heme availability, signaling, and HO function.

The oxidation state of LH in eukaryotic cells has been a long-standing unresolved mystery. LH was first characterized *in situ* in live human cell lines with genetically encoded FRET-based heme sensors (68). In these seminal studies, only the ferric heme affinities of the sensors were determined and estimates of buffered-free heme concentrations based on sensor heme occupancy were by default assumed to be ferric. These studies found free heme to be ∼20 nM in a variety of nonerythroid human cell lines, including HEK293 cells. Studies using heme-peroxidase activity-based reporters, which bind ferric heme in its resting state, found free heme to be as high as ∼400 nM in HEK293 cell lysates (73). These estimates may be higher because heme measurements were performed in disrupted cell extracts, and cell lysis may have resulted in heme dissociation from certain heme-binding sites. Interestingly, unlike in HEK293 cells, in *S. cerevisiae*, heme occupancies for the high affinity heme sensor, HS1, was found to be 100% and the lower affinity sensor, HS1-M7A, was 30 to 50% heme loaded. At the time of these studies, it was assumed that LH was largely reduced on the basis of the ferrochelatase-catalyzed insertion of ferrous iron into protoporphyrin IX to make reduced heme. The heme occupancy of HS1-M7A coupled with its *K*_D_^II^ value of 25 nM led to estimates of free heme being ∼25 nM (if LH was primarily reduced) in yeast. Altogether, before this study, there were no methods to parse LH oxidation state.

In the present report, we exploited HO-2 selectivity for ferric heme over ferrous heme (94) and perturbations to its expression by silencing or overexpression to infer that LH is largely oxidized (Fig. 6). Labile heme being oxidized is consistent with the observation that DNA and RNA guanine quadruplexes, which have nM dissociation constants for ferric heme, also appear to bind and regulate heme availability in human cell lines (67, 71). In addition, the HS1 heme occupancy and corresponding buffered-free heme concentration observed herein is identical to a recent study in HEK293 cells using a peroxidase-based heme reporter, which is expected to preferentially bind ferric heme (69). Moreover, oxidized LH is also consistent with previously reported roles of oxidized heme in signaling. For instance, ferric, but not ferrous, heme is required for RNA-binding protein DGCR8 for primary microRNA processing (95). The nuclear receptor Rev-Erbβ can sense and bind ferric heme, which can then act as a sensor for CO or NO by coupling gas binding to heme reduction (96). In addition, ferric heme was demonstrated to regulate ATP-dependent potassium channels (97).

Although LH is largely oxidized in HEK293 cells, it may vary between different cell types and be highly dynamic and responsive to various stimuli. The Fe(III)/Fe(II) redox couple is linked to a number of factors, including access to certain cellular reductants or oxidants (98), ligand binding to the heme iron center, for example, CO or NO (96), and allosteric protein conformational changes (99). Exactly how these factors conspire to affect steady-state LH oxidation state, its dynamics, and role in metabolism and physiology are not known and may be elucidated by the future development of oxidation-state specific heme sensors or chelators.

Our results suggesting that endogenous LH or free heme is too limiting in some cell types to support substantial HO activity has implications for the function of HO-2, HO-1, and emerging roles for heme catabolites, for example, CO, biliverdin, and bilirubin in physiology. First, our results explain why overexpression of catalytically inactive HO-2 alleles are cytoprotective against peroxide stress (76). It likely does so by sequestering heme, presumably to prevent heme-dependent peroxidase or Fenton reactions that are damaging to cells. By extension, we suggest that nonheme stressors, for example, reactive oxygen species, metals, and various xenobiotics, that induce HO-1 expression may result in cytoprotection due to HO-1 mediated heme sequestration because endogenous LH or free heme may be too limiting to promote significant rates of heme degradation in many cell types. The heme sequestration mechanism for heme detoxification may also occur if electron delivery to the HOs *via* CPR is limiting. During heme stress and provided that there is sufficient CPR and NADPH, inducible HO-1 and constitutive HO-2 are expected to enzymatically degrade and detoxify excess heme, with their relative contributions being dictated by their respective expression levels.

The ratio of HO-2 to HO-1 protein expression is highly variable across different cell lines and tissues, spanning 5000 (frontal cortex) to 0.03 (spleen) (Fig. S6) (100). Moreover, HO-1 can be induced by as much as 100-fold in response to various stressors (101). Thus, cells occupy a position along an expansive continuum between their reliance on HO-1 and HO-2. The cell line used in this study, HEK293 cells, has a HO-2 to HO-1 ratio of 2 (100, 102), explaining why silencing of HO-2 still results in measurable bilirubin levels. In most cell types, it is not known if free heme or LH is too limiting to support a significant amount of HO activity in the absence of excess heme stress, as it is in HEK293 cells, raising the intriguing possibility that HO-2, and possibly HO-1, have other roles in heme homeostasis beyond heme degradation. In cell lines that express both HO-1 and HO-2, it is possible their roles in regulating heme homeostasis diverge, as described in microbes such as *Staphylococcus aureus* that express dual HO isoforms with distinct roles in regulating heme synthesis and degradation (103).

Consistent with a potential role for HO-2 in controlling access to heme, it was recently demonstrated that HO-2 is degraded by the lysosomal pathway during heme deficiency (64), presumably to increase heme availability. A role for HO-2 in regulating heme availability needs to be explored further and could represent a mechanism for heme sparing during periods of heme limitation. On the other hand, there may be certain cell types in which endogenous LH or free heme is much higher than HEK293 cells, for example, cells of the brain and testes, where HO-2 is most highly expressed. Indeed, prior work suggests that, in the brain, HO-2-derived heme catabolites can provide protection against oxidative stress and other injuries (31, 104, 105). Our future studies will involve profiling LH in multiple cell types with varying levels of HO-2 and HO-1 expression to better assess HO function with respect to its heme degrading and buffering functions.

Carbon monoxide, biliverdin, and bilirubin, products of heme degradation, have emerged as key metabolites with distinct physiological roles (32, 47, 48, 49, 50, 51, 52, 53, 54, 55). The only source of these metabolites is from heme degradation *via* the HO system. Our studies in HEK293 cells finding that LH is limiting suggests that heme catabolite signaling in many cell types must be coupled to substantially increased heme synthesis, redistribution of LH, or influx of heme. In *S. cerevisiae*, NO signaling (63) and heavy metal stress (62) was found to increase LH, suggesting that other physiological inputs may augment LH to support heme catabolite production. In addition, given the identification of mammalian heme importers and exporters, cells may communicate *via* heme, ultimately transducing heme signals to heme catabolites *via* the HOs to regulate physiology and metabolism.

Most interestingly, we found that HO-2 overexpression limits heme availability in the cytosol and nucleus, but not the mitochondria (Fig. 4*D*). These data indicate that the mitochondrial LH pool is insulated from the generation of a cytosolic heme sink. In contrast, if mitochondrial LH is populated with exogenously derived heme, by supplementing cells cultured in HD + SA media with 6 μM hemin chloride, overexpression of HO-2 does reduce mitochondrial LH (Fig. S7). Together, these data suggest that mitochondrially produced heme flows in only one direction, from the inside out and not vice versa. In contrast, exogenously supplied heme can gain access to the mitochondria, but in a manner that is impacted by a cytosolic heme sink. These data are consistent with prior findings that exogenously supplied and endogenously synthesized heme are trafficked through separate pathways (73). Indeed, we found herein that although 350 μM ALA and 50 μM heme supplementation led to similar total intracellular heme levels, only the latter was able to populate the lower affinity HS1-M7A heme sensor (Fig. 2, *C* and *D*), further highlighting possible differences in the trafficking, bioavailability, and oxidation state of exogenous and endogenous heme.

Altogether, our studies demonstrate that, in some cell types, constitutive HO-2 does not catalyze heme degradation at rates fast enough to observe perturbations to total heme or bilirubin levels upon silencing or overexpression of HO-2 due to heme being too limiting a substrate. Instead, we suggest that HO-2 may have alternative roles in regulating heme availability by acting as a buffer or reservoir when available heme is below its heme *K*_M_ of 400 nM and on the order of its *K*_D_ of 3.6 nM. As available heme levels approach or exceed the *K*_M_ value, HO-2 may contribute substantially toward heme degradation along with inducible HO-1. The identification of a heme buffering function for HO-2 in regulating heme availability places it with other factors, such as glyceraldehyde phosphate dehydrogenase (GAPDH) (63, 70, 106, 107), rRNA and DNA guanine quadruplexes (67, 71), and mitochondrial-ER contacts sites (66), that affect intracellular heme availability and distribution. Future work is required to firmly establish the physiological context and consequences of HO-2 mediated regulation of heme availability independent of its role in heme degradation.

## Experimental procedures

### Materials

Fetal bovine serum (FBS) (Catalog # 89510-188), Dulbecco’s Modified Eagle’s Medium (DMEM) (Catalog # L0102-0500), Trypsin 0.25% w/v (Catalog # 45000-664), ALA (Catalog # BT143115-2G), ascorbic acid, cell culture grade dimethylsulfoxide (DMSO), oxalic acid, Dulbecco’s Phosphate Buffered Saline (DPBS), and LiCOR Intercept tris-buffered saline blocking buffer (Catalog # 103749-018) were purchased from VWR/Avantor. Opti-MEM (Catalog # 31-985-070) was purchased from Fischer Scientific. Lipofectamine LTX and PLUS reagent (Catalog # 15338100), Lipofectamine RNAiMax (Catalog # 13778075), and a mounting reagent with 4′,6-diamidino-2-phenylindole were purchased from Invitrogen. Succinylacetone (Catalog # D1415) was purchased from Sigma Aldrich. Digitonin and hemin chloride were acquired from Calbiochem. The following primary and secondary antibodies were used: rabbit polyclonal HO-2 antibody (Abcam ab90515); mouse monoclonal GAPDH antibody (Sigma 8795); rabbit polyclonal HO-1 antibody (Enzo Life Sciences BML-HC3001-0025); mouse monoclonal calnexin antibody (Invitrogen MA3-027); mouse monoclonal ubiquitin antibody (P4D1) (Santa Cruz Biotechnology sc-8017); anti-FLAG M2 magnetic bead (Sigma Aldrich M8823); goat anti-rabbit secondary antibody (Biotium 20064); and goat anti-mouse secondary antibody (Invitrogen SA5-35521).

### Plasmid constructs and mutagenesis

The previously described FLAG-tagged WT and mutant HO2 constructs were subcloned into the pcDNA3.1 plasmid and driven by the CMV promoter (64). For untargeted cytosolic expression of human codon optimized heme sensors, HS1, HS1-M7A, and HS1-M7A,H102A, the heme sensors were expressed using the pcDNA3.1 plasmid and driven by the CMV promoter, as previously described (63, 67). For heme sensing experiments involving mitochondrial matrix and nuclear targeting of HS1, the constructs were generated *via* Gateway Cloning (Invitrogen Life Technologies) by PCR amplifying the hHS1 constructs with primers containing 5′-attB1 (G GGG ACA AGT TTG TAC AAA AAA GCA GGC T) and 3′-attB2 (GGG GAC CAC TTT GTA CAA GAA AGC TGG GT) sequences. The purified PCR fragments were cloned into pDONR221 vector *via* attB x attP recombination reaction. The resulting entry clones were subjected to LR recombination reaction with pEF5/FRT/V5-DEST vector to obtain the expression plasmids. The mitochondrial and nuclear targeting sequences used were from subunit VIII of human COXIV, MSVLTPLLLRGLTGSARRLPVPRAKIHSL, and a nuclear localization sequence, MDPKKKRKVDPKKKRKV (73). Sequences and plasmid maps are provided in supporting information. All plasmids were confirmed by Sanger sequencing.

### Cell culture, growth conditions, plasmid transfections, and RNA interference

HEK293 cells used in this study were previously described and obtained from American Type Culture Collection (63, 67). HEK293 cells were grown in regular media – (DMEM, with 4.5 g/l glucose and without L-glutamine and sodium pyruvate, supplemented with 10% v/v FBS (heat inactivated) in T75 flasks (Greiner) (Regular Media). The cells were split using 0.25% w/v Trypsin.

To deplete heme, the cells were cultured with 500 μM SA in HD media - DMEM containing 10% v/v heme depleted FBS - for 48 to 72 h before harvesting (67, 73, 95, 108). To increase heme, the cells were cultured with 350 μM ALA or between 1 and 50 μM hemin chloride in regular or HD media for 20 to 24 h. Heme-depleted FBS was generated by incubating it with 10 mM ascorbic acid in a 37 °C shaking incubator at 200 RPM for 8 h (67, 73, 95, 108). Loss of heme was monitored by measuring the decrease in Soret band absorbance at 405 nm; typically the initial Abs_405nm_ was 1.0 and the final Abs_405nm_ was 0.5 (67, 73, 95, 108). Eighty milliliters of ascorbate-treated FBS was then dialyzed against 3 l of 1× PBS, pH 7.4, three times for 24 h each at 4 °C, using a 2000 MWCO membrane (Spectra/Por 132625). Dialyzed FBS was then filter sterilized with a 0.2 μm polyethersulfone filter (VWR 28145-501) and syringe (VWR 53548-010).

For heme sensor transfections, 1/24 of the HEK293 cells from a confluent T75 flask were used to seed 2 ml cultures in each well of polystyrene-coated sterile 6-well plates (Greiner). Once the cells were 30% to 50% confluent, 1 to 2 days after seeding, the media was changed to 2 mls of fresh regular or HD+SA media 30 to 60 min before transfection. For each well to be transfected, 100 μl of a plasmid transfection master mix was added. The master mix was prepared by mixing, in order, 600 μl OptiMEM (100 μl per well up to 600 μl), 2 μg of heme sensor plasmid, a volume of Lipofectamine Plus Reagent equal in μl to the μg of plasmid DNA used, and a volume of Lipofectamine LTX transfection reagent that is double the volume of the Lipofectamine Plus Reagent. Before adding the transfection mixture to each 6-well plate, the master mix was mixed by gentle pipetting and allowed to incubate at room temperature (25 °C) for 5 min. Forty eight hours after transfection, the media was changed to fresh regular or HD+SA media and supplemented with the appropriate concentration of ALA or hemin chloride as indicated. The cells were then cultured for an additional 20 to 24 h before harvesting for various analyses, including measurements of LH, total heme, and bilirubin, as well as immunoblotting.

If cotransfecting the heme sensors with the HO2 plasmids, the same procedure was followed as above, with the exception that 2 μg of the HO-2 plasmid DNA was added at the same time as the heme sensor plasmid, and twice the volumes of the Lipofectamine Plus and LTX reagents were used.

For cytosolic, mitochondrial, or nuclear targeted pEF-hHS1 in which HO-2 plasmids were not cotransfected, PolyJET was used in plasmid transfections. The procedure is identical to that described above, except that 100 μl of a PolyJET transfection reagent master mix was added to each well in a 6-well plate. Per well, the master mix consisted of 50 μl of DPBS with 1 to 2 μg of plasmid DNA and 50 μl of DPBS with 4 to 8 μl of PolyJET and was incubated at room temperature for 10 to 15 min before adding to the cells. Twenty four to forty hours posttransfection posttransfection, the media was changed and cells were then cultured for an additional 20 to 24 h with ALA or hemin chloride as indicated before harvesting for LH and total heme measurements.

To silence HO-2 expression in HEK293 cells, we used siRNA against HO-2. HEK293 cells were plated in polystyrene-coated sterile 6-well plates (Greiner) and cultured in regular media as described above. At 10 to 30% confluency, the cells were transfected with siHMOX2 (Dharmacon L-009630-00-0005) or nontargeting pool (Dharmacon D-001810-10-05) siRNA using Lipofectamine RNAi Max (Thermofisher). Briefly, for each well to be transfected, 9 μl of Lipofectamine RNAiMax was added to 150 μl of OptiMEM and 3 μl of 10 μM siRNA to 150 μl OptiMEM. The two solutions were mixed 1:1 and allowed to incubate at room temperature for 5 min. After incubation, 250 μl of the transfection solution was added to the 2 ml cultures. After 24 h, the HO-2 silencing protocol was repeated, and the cells were cultured for an additional 48 h. After 72 h of control or siRNA treatment, the cells were harvested for analysis.

To transfect the heme sensor plasmids into HO-2 silenced cells, the pEF5-hHS1 plasmid was transfected using the Lipofectamine LTX reagents as described above after 72 h of control or siRNA treatment. After transfecting the heme sensor, the cells were cultured for an additional 72 h in either HD+SA or regular media. To induce heme excess, the cells cultured in regular media were treated with 350 μM ALA for 24 h before harvesting.

### Heme sensor calibration

To determine sensor heme occupancy, *R*_min_ and *R*_max_ was determined by depleting heme from cells or saturating the sensor with heme, respectively (Equation 1). Heme depletion was conducted by growing parallel cultures in HD + SA media for 3 days, as described above. To saturate the sensor with heme, cells from a well of a 6-well dish were resuspended in 1 ml of “sensor calibration buffer”, transferred to a microfuge tube, and incubated in a 30 °C water bath for 30 min. After this, the cells were pelleted at 400*g* for 4 min at 4 °C, the media aspirated, the cell pellet washed with 1 ml PBS at room temperature once, and finally resuspended in 500 μl PBS containing 1 mM ascorbate before being analyzed by flow cytometry. The sensor calibration buffer consisted of 1 ml of serum-free DMEM (prewarmed at 37 °C), 40 μM digitonin (from a 1 mg/ml stock solution in PBS), 100 μM hemin chloride (from a 10 mg/ml DMSO stock), and 1 mM ascorbate (from a 100 mM stock solution in PBS). The sensor calibration buffer saturates cytosolic and nuclear hHS1 and partially saturates mitochondrial hHS1. An alternative method to saturate hHS1 in all compartments tested, including the mitochondria, involved culturing cells in 350 μM ALA for 24 h before fluorescence analysis.

### Flow cytometry

HEK293 cells were transfected and cultured as described above in 6-well polystyrene-coated plates. Before analysis, the cells were washed and resuspended in 1 ml of 1× PBS and transferred to a 1.5 ml microfuge tube and pelleted at 400*g* and 4 °C for 4 min. Supernatant was decanted and the cells were resuspended in 500 μl 1× PBS. Cell suspension was filtered through 35 μm nylon filter cap on 12 × 75 mm round bottom tubes (VWR/Falcon 21008-948). Flow cytometric measurements were performed using a BD LSR Fortessa, BD LSR II, or BD FACS Aria Illu flow cytometers equipped with an argon laser (ex 488 nm) and yellow-green laser (ex 561 nm). Enhanced green fluorescent protein was excited using the argon laser and was measured using a 530/30 nm bandpass filter, whereas mKATE2 was excited using the yellow-green laser and was measured using a 610/20 nm bandpass filter. Data evaluation was conducted using FlowJo v10.4.2 software. Single cells were gated by size (FSC *versus* SSC) and only mKATE2 positive cells that had median mKATE2 fluorescence were selected for ratiometric analysis, which typically corresponded to ∼5000 cells.

### Bilirubin measurements

HEK293 cells were transfected and cultured as described above in 6-well polystyrene-coated plates. Before analysis, the cells were washed and resuspended in 1 ml of 1× PBS and transferred to a 1.5 ml microfuge tube and pelleted at 400*g* 4 °C for 4 min. Supernatant was decanted and the cells were resuspended in 1 ml 1× PBS and counted using a TC20 Automated Cell Counter from BioRad. 1 × 10^6^ cells were pelleted and resuspended in Lysis Buffer (10 mM NaPi 50 mM NaCl, 1% v/v Triton X-100, 5 mM EDTA, 1 mM PMSF, and 1× Protease Arrest (G-Bioscience 786-437)), then lysed by freeze-thawing using 3 cycles of 10 min at −80 °C and 15 min at RT. Lysates were clarified by centrifugation at 21,100*g* in a table-top microfuge at 4 °C for 10 min 1% v/v DMSO was added to the cell lysate to solubilize the bilirubin. Total bilirubin was quantified using the Bilirubin Assay kit from Sigma-Aldrich exactly as described in the manufacturer’s protocol (MAK126). A standard curve was generated from bilirubin standards made up in cell lysis buffer with 1% v/v DMSO. Bilirubin concentrations were normalized to cell number, and a cellular concentration was determined by assuming a cell volume of 1.2 pl (109).

### Total heme measurements

Measurements of total heme were accomplished using a fluorometric assay designed to measure the fluorescence of protoporphyrin IX upon the release of iron from heme, as previously described (62, 67, 110). After the harvesting of HEK293 cells as described above, the cells were counted using an automated TC20 cell counter (BioRad). At least 500,000 cells were resuspended in 400 μls of 20 mM oxalic acid overnight at 4 °C protected from light. After the overnight incubation, an equal volume of 2 M warm oxalic acid was added to give a final oxalic acid concentration of 1M. The oxalic acid cell suspensions were split, with half the cell suspension transferred to a heat block set at 100 °C and heated for 30 min and the other half of the cell suspension kept at room temperature (∼25 °C) for 30 min. All suspensions were centrifuged for 3 min on a table-top microfuge at 21,000*g*, and the porphyrin fluorescence (ex: 400 nm, em: 620 nm) of 200 μl of each sample was recorded on a Synergy Mx multi-modal plate reader using black Greiner Bio-one flat bottom fluorescence plates. Heme concentrations were calculated from a standard curve prepared by diluting 2.5 to 200 nM hemin chloride stock solutions in 0.1 M NaOH into oxalic acid solutions prepared the same way as for the cell samples. To calculate heme concentration, the fluorescence of the unboiled sample (taken to be the background level of protoporphyrin IX) is subtracted from the fluorescence of the boiled sample (taken to be the free porphyrin generated upon the release of heme iron). The cellular concentration of heme is determined by dividing the moles of heme determined in this fluorescence assay and dividing by the number of cells analyzed, giving moles of heme per cell, and then converting to a cellular concentration by dividing by the volume of a HEK293 cells, assumed to be 1.2 pl (109).

### Immunoblotting

Cells were harvested from a 6-well plate in 1× PBS and pelleted at 21,100*g* at 4 °C for 1 min and resuspended in lysis buffer (10 mM NaPi 50 mM NaCl, 1% v/v Triton X-100, 5 mM EDTA, 1 mM PMSF, and 1× Protease Arrest (G-Bioscience 786-437)). The cells were lysed by freeze-thawing using 3 cycles of 10 min at −80 °C and 15 min at RT. The lysates were clarified by centrifugation at 21,100*g* in a table-top microfuge at 4 °C for 10 min. Lysate protein was quantified by Bradford Assay. Typically, 15 to 30 μg of lysate protein was prepared in a final volume of 20 μl loading buffer, with 1× SDS loading dye in lysis buffer (5× SDS loading dye consists of 312.5 mM Tris–HCl, pH 6.8, 50% v/v glycerol, 346 mM SDS, 518 mM DTT, and 7.5 mM Bromophenol blue). Samples were boiled at 100 °C for 5 min in a heat block. The lysate samples were loaded onto a 14% w/v Tris–glycine SDS-PAGE gel (Invitrogen XP00140BOX) and electrophoresed at a constant current of 20 mA for 1.5 h. Gels were transferred onto 0.2 um nitrocellulose membranes (Biotrace) overnight, but no longer than 2 days, using the XCell II Blot Module (Invitrogen) for a wet tank transfer. The membranes were blocked with Intercept tris buffered saline Blocking Buffer (LICOR 927-50000). Heme oxygenase-2 and HO-1 were probed with a 1:1000 dilution of rabbit anti HO-2 antibody (Abcam 90515) or 1:1000 rabbit anti HO-1 (Enzo Life Sciences BML-HC3001-0025) at 4 °C overnight, respectively. GAPDH was probed with a 1:4000 dilution of mouse anti GAPDH (Sigma 8795) for 1 h at room temperature. Goat anti-rabbit secondary antibody conjugated to a 700 nm fluorophore (Biotium 20064) or goat anti-mouse secondary antibody conjugated to a 800 nm fluorophore (Invitrogen SA5-35521) was used at a dilution of 1:10,000 for 1 h at room temperature. After application of primary and secondary antibodies, the membranes were washed with 1× Tris Buffered Saline-Tween (24.7 mM Tris-HCl pH 7.4 13.6 mM NaCl, 2.6 mM KCl 0.1% v/v Tween 20) two times for 10 min at room temperature. The membranes were imaged using a LiCOR Odyssey Clx imager.

### Immunofluorescence microscopy

The experimental approach for immunofluorescence microscopy are adapted from previous literature (64). Briefly, HEK293 cells were cultured on poly-lysine coated coverslips, transfected with plasmids expressing FLAG-tagged HO2 and its variants, washed with phosphate buffered saline (PBS), and fixed with 4% w/v paraformaldehyde before immunostaining. Immunofluorescence microscopy samples were permeabilized with 1× PBS containing 0.2% v/v Triton X-100 and stained with corresponding antibodies. Sample images were acquired on an Olympus IX81 microscope with a Yokogawa CSU-W1 spinning disk confocal scanner unit, an Olympus PlanApo 60× 1.42 NA objective, and a Hamamatsu ImagEMX2-1K EM-CCD digital camera.

### Sensor microscopy

For heme sensor imaging, 1 day before transfection, HEK293 cells were seeded at a density of 1 × 10^5^ cells per well on sterile coverslips in 6-well plates. Each well of the cells was transfected with 1 μg plasmid DNA and 3 μl PolyJet (SignaGen, CAT#SL100688) according to the manual instructions. The media was changed to fresh regular or HD+SA media immediately before transfection and changed again after 24 h to regular or HD+SA media and, if ALA or hemin chloride was to be added, it would be added at this time. The cells were cultured for an additional 18 h, fixed in 4% w/v paraformaldehyde for 1 h at room temperature, stained with 4′,6-diamidino-2-phenylindole, and mounted with Prolong Antifade (Invitrogen). Fluorescence images were taken on a Leica DM IRE2 microscope using 63× oil immersion lens.

### Statistics

All experiments were conducted with at least three biological replicates and conducted in at least two independent trials. All ANOVA were conducted using GraphPad Prism software version 9 and considered to be significant at the level of *p* < 0.05 (GraphPad Software, Inc).

## Data availability

All data is available in the article or its supporting information.

## Supporting information

This article contains supporting information (85, 86, 100, 102).

## Conflict of interest

The author declares that they have no conflicts of interest with the contents of this article.
