# Supplementary material for: Heme oxygenase-2 (HO-2) binds and buffers labile ferric heme in human embryonic kidney cells
Source: J Biol Chem. 2021 Dec 29;298(2):101549. doi: 10.1016/j.jbc.2021.101549 (PMC8808069; doi:10.1016/j.jbc.2021.101549)
Supplement: Supplemental Figures S1–S9 [file mmc1.pdf]

Supporting Information

Heme oxygenase-2 (HO-2) binds and buffers labile ferric heme in human embryonic kidney cells

David A. Hanna, Courtney M. Moore, Liu Liu, Xiaojing Yuan, Iramofu M. Dominic, Angela S. Fleischhacker, Iqbal Hamza, Stephen W. Ragsdale, Amit R. Reddi

Table of Contents

1. Heme Sensor Plasmid Maps and Sequences for pEF-HS1 Constructs……………………….S1
2. Supporting Figures (S1 – S9)…………………………………………………………………………….S16
3. Supporting References……………………………………………………………………………………..S25

1. Heme Sensor Plasmid Maps and Sequences for pEF5-HS1 Constructs

hHS1

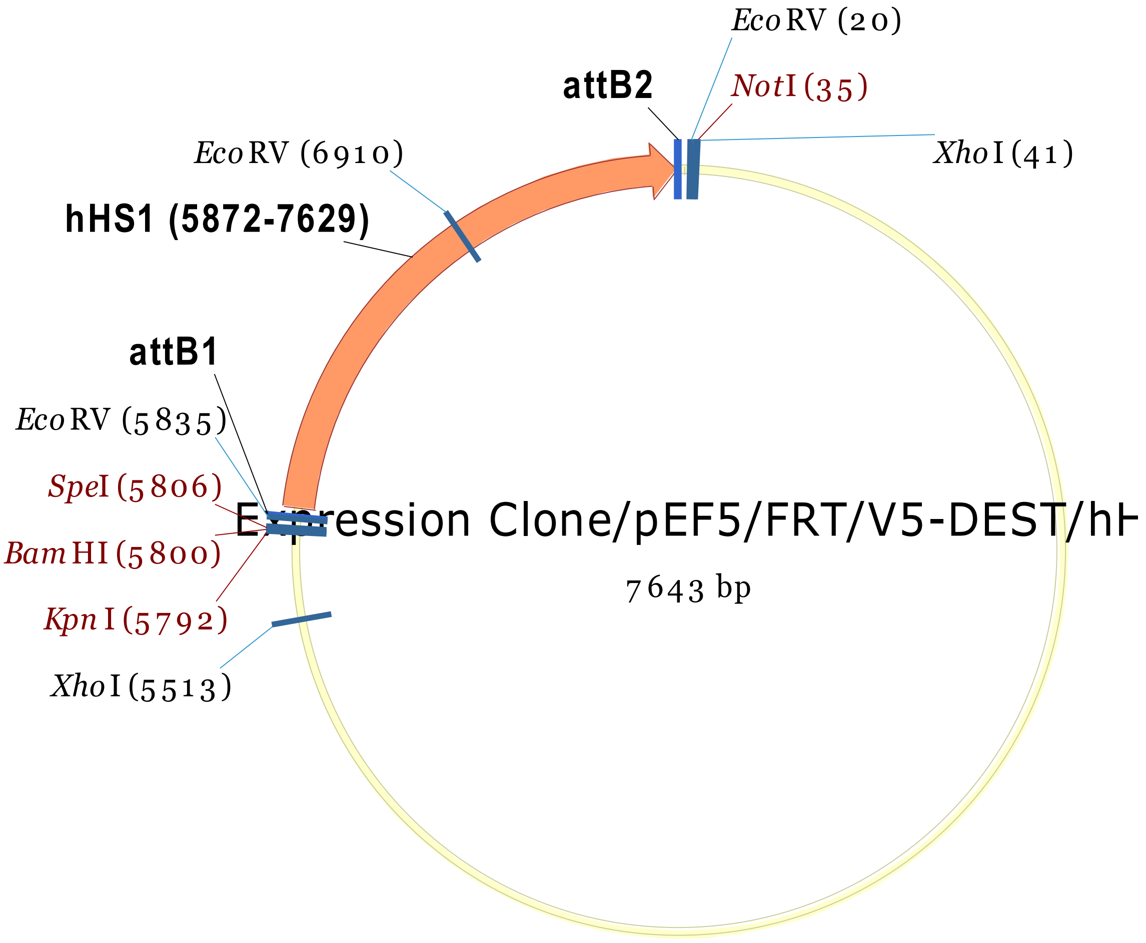

hHS1

ATGCACATGGTCAGCGAGCTGATCAAGGAAAACATGCACATGAAACTGTACATGGAGGGGACTGTGAA
CAATCACCATTTCAAATGCACCTCCGAGGGCGAAGGGAAGCCCTACGAGGGCACACAGACTATGAGGA
TCAAGGCAGTGGAGGGAGGACCACTGCCATTCGCCTTTGACATTCTGGCTACCTCATTCATGTACGGCA
GCAAAACCTTCATCAATCACACTCAGGGGATTCCCGACTTCTTTAAGCAGTCTTTCCCTGAAGGCTTTACT
TGGGAGCGAGTGACCACATACGAGGATGGAGGCGTCCTGACCGCCACACAGGACACAAGTCTGCAGGA
TGGCTGTCTGATCTATAACGTGAAGATTCGCGGGGTCAACTTTCCCAGTAATGGACCTGTGATGCAGAA
GAAAACCCTGGGATGGGAGGCTTCAACTGAAACCCTGTACCCAGCAGACGGAGGACTGGAGGGACGA
GCAGATATGGCTCTGAAACTGGTGGGCGGGGGACACCTGATCTGCAACCTGAAGACTACCTATCGGTCC
AAGAAACCTGCTAAGAATCTGAAAATGCCAGGCGTGTACTATGTGGACCGGAGACTGGAGAGAATTAA
GGAAGCAGATAAAGAGACCTACGTGGAGCAGCACGAAGTGGCTGTCGCACGATATTGTGACCTGCCTT
CTAAACTGGGCCATCGGGGCGGGTCTATGGTGAGTAAGGGCGAGGAACTGTTCACAGGGGTGGTCCCA

ATCCTGGTGGAACTGGACGGCGATGTCAATGGGCACAAGTTCAGCGTGTCCGGAGAGGGAGAAGGGG
ACGCAACCTTTGGAGGCAGCGCCGACCTGGAAGATAATATGGAGACACTGAACGATAATCTGAAAGTG
ATCGAGAAAGCCGACAACGCCGCTCAGGTCAAGGATGCTCTGACTAAAATGAGGGCAGCCGCTCTGGA
TGCACAGAAAGCCACCCCCCCTAAGCTGGAAGACAAATCACCTGATAGCCCAGAGATGAAGGACTTCCG
CCACGGATTTGATATCCTGGTCGGCCAGATTGACGATGCTCTGAAGCTGGCAAATGAAGGCAAGGTGA
AAGAGGCACAGGCAGCCGCTGAGCAGCTGAAAACAACTAGGAACGCCTACCATCAGAAGTATCGCGGG
GGAAAGCTGACACTGAAATTCATCTGCACCACAGGCAAGCTGCCCGTGCCCTGGCCAACTCTGGTCACT
ACCCTGGGATACGGCGTGCAGTGTTTTTCCCGCTATCCAGACCACATGAAGCAGCATGATTTCTTTAAAT
CTGCCATGCCCGAAGGCTACGTGCAGGAGAGAACCATCTTCTTTAAGGACGATGGAAACTATAAAACAA
GGGCTGAAGTGAAGTTCGAGGGAGACACTCTGGTCAACCGCATCGAACTGAAGGGCATTGACTTTAAA
GAGGATGGAAATATTCTGGGCCACAAGCTGGAATACAACTATAATAGCCATAACGTGTACATCATGGCC
GATAAGCAGAAAAACGGCATTAAGGTCAATTTCAAAATCCGGCACAATATTGAGGACGGGAGCGTGCA
GCTGGCCGATCATTACCAGCAGAACACCCCAATCGGGGACGGACCAGTGCTGCTGCCCGATAATCACTA
TCTGTCCACACAGTCTGCCCTGAGTAAGGACCCTAACGAAAAAAGAGATCACATGGTGCTGCTGGAGTT
TGTCACCGCAGCCGGGATTACACTGGGAATGGACGAGCTGTACAAGTGA

NLS-hHS1

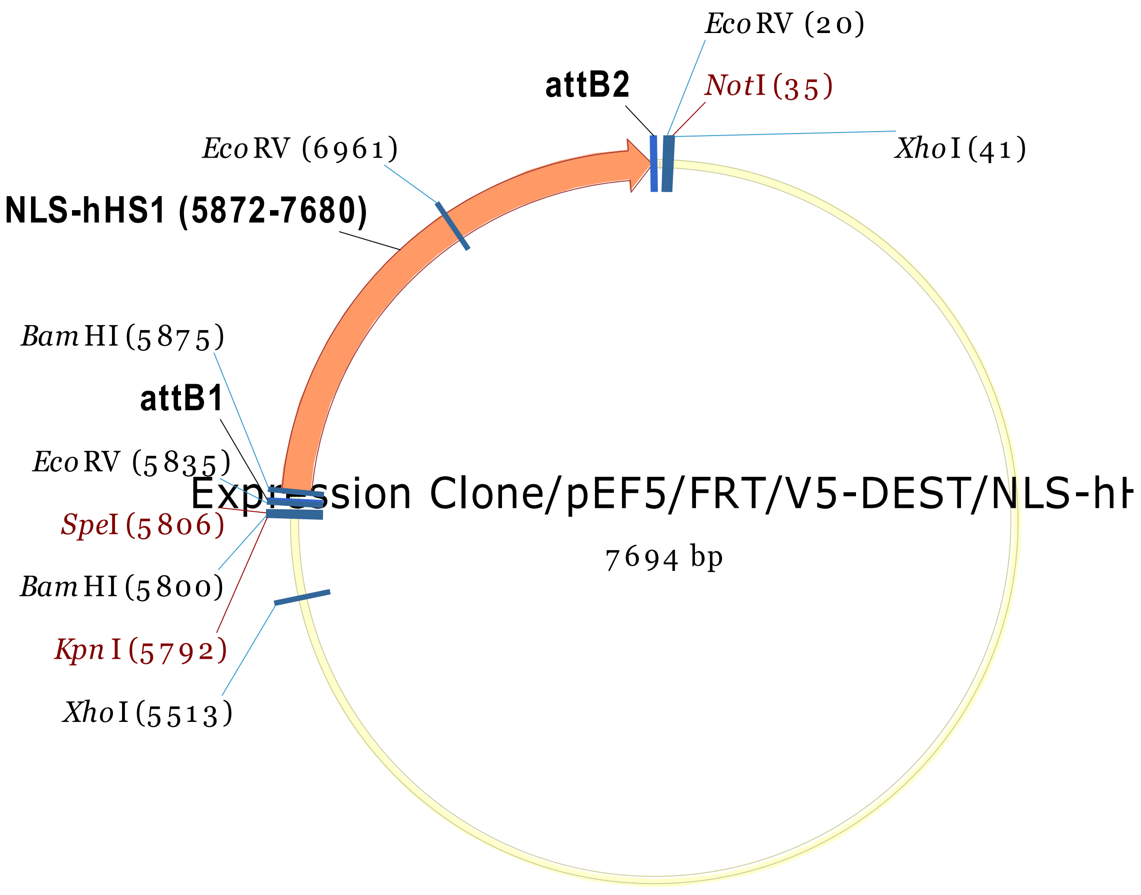

NLS-hHS1

ATGGATCCAAAAAAGAAGAGAAAGGTAGATCCAAAAAAGAAGAGAAAGGTAATGCACATGGTCAGCG
AGCTGATCAAGGAAAACATGCACATGAAACTGTACATGGAGGGGACTGTGAACAATCACCATTTCAAAT
GCACCTCCGAGGGCGAAGGGAAGCCCTACGAGGGCACACAGACTATGAGGATCAAGGCAGTGGAGGG
AGGACCACTGCCATTCGCCTTTGACATTCTGGCTACCTCATTCATGTACGGCAGCAAAACCTTCATCAATC
ACACTCAGGGGATTCCCGACTTCTTTAAGCAGTCTTTCCCTGAAGGCTTTACTTGGGAGCGAGTGACCAC
ATACGAGGATGGAGGCGTCCTGACCGCCACACAGGACACAAGTCTGCAGGATGGCTGTCTGATCTATA
ACGTGAAGATTCGCGGGGTCAACTTTCCCAGTAATGGACCTGTGATGCAGAAGAAAACCCTGGGATGG
GAGGCTTCAACTGAAACCCTGTACCCAGCAGACGGAGGACTGGAGGGACGAGCAGATATGGCTCTGAA
ACTGGTGGGCGGGGGACACCTGATCTGCAACCTGAAGACTACCTATCGGTCCAAGAAACCTGCTAAGA
ATCTGAAAATGCCAGGCGTGTACTATGTGGACCGGAGACTGGAGAGAATTAAGGAAGCAGATAAAGAG
ACCTACGTGGAGCAGCACGAAGTGGCTGTCGCACGATATTGTGACCTGCCTTCTAAACTGGGCCATCGG
GGCGGGTCTATGGTGAGTAAGGGCGAGGAACTGTTCACAGGGGTGGTCCCAATCCTGGTGGAACTGGA
CGGCGATGTCAATGGGCACAAGTTCAGCGTGTCCGGAGAGGGAGAAGGGGACGCAACCTTTGGAGGC
AGCGCCGACCTGGAAGATAATATGGAGACACTGAACGATAATCTGAAAGTGATCGAGAAAGCCGACAA
CGCCGCTCAGGTCAAGGATGCTCTGACTAAAATGAGGGCAGCCGCTCTGGATGCACAGAAAGCCACCC
CCCCTAAGCTGGAAGACAAATCACCTGATAGCCCAGAGATGAAGGACTTCCGCCACGGATTTGATATCC
TGGTCGGCCAGATTGACGATGCTCTGAAGCTGGCAAATGAAGGCAAGGTGAAAGAGGCACAGGCAGC
CGCTGAGCAGCTGAAAACAACTAGGAACGCCTACCATCAGAAGTATCGCGGGGGAAAGCTGACACTGA
AATTCATCTGCACCACAGGCAAGCTGCCCGTGCCCTGGCCAACTCTGGTCACTACCCTGGGATACGGCGT
GCAGTGTTTTTCCCGCTATCCAGACCACATGAAGCAGCATGATTTCTTTAAATCTGCCATGCCCGAAGGC
TACGTGCAGGAGAGAACCATCTTCTTTAAGGACGATGGAAACTATAAAACAAGGGCTGAAGTGAAGTTC
GAGGGAGACACTCTGGTCAACCGCATCGAACTGAAGGGCATTGACTTTAAAGAGGATGGAAATATTCT
GGGCCACAAGCTGGAATACAACTATAATAGCCATAACGTGTACATCATGGCCGATAAGCAGAAAAACG
GCATTAAGGTCAATTTCAAAATCCGGCACAATATTGAGGACGGGAGCGTGCAGCTGGCCGATCATTACC
AGCAGAACACCCCAATCGGGGACGGACCAGTGCTGCTGCCCGATAATCACTATCTGTCCACACAGTCTG
CCCTGAGTAAGGACCCTAACGAAAAAAGAGATCACATGGTGCTGCTGGAGTTTGTCACCGCAGCCGGG
ATTACACTGGGAATGGACGAGCTGTACAAGTGA

Mito-hHS1

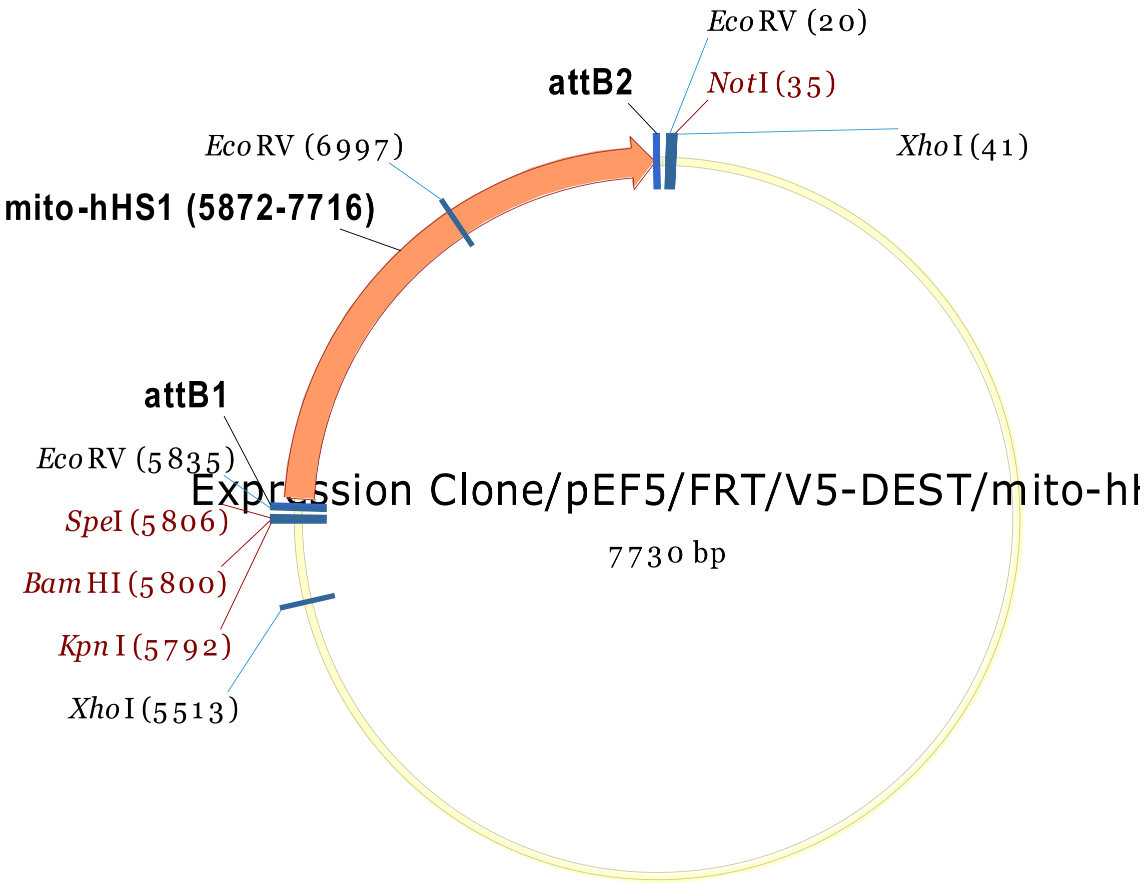

Mito-hHS1

ATGTCCGTCCTGACGCCGCTGCTGCTGCGGGGCTTGACAGGCTCGGCCCGGCGGCTCCCAGTGCCGCGC
GCCAAGATCCATTCGTTGATGCACATGGTCAGCGAGCTGATCAAGGAAAACATGCACATGAAACTGTAC
ATGGAGGGGACTGTGAACAATCACCATTTCAAATGCACCTCCGAGGGCGAAGGGAAGCCCTACGAGGG
CACACAGACTATGAGGATCAAGGCAGTGGAGGGAGGACCACTGCCATTCGCCTTTGACATTCTGGCTAC
CTCATTCATGTACGGCAGCAAAACCTTCATCAATCACACTCAGGGGATTCCCGACTTCTTTAAGCAGTCTT
TCCCTGAAGGCTTTACTTGGGAGCGAGTGACCACATACGAGGATGGAGGCGTCCTGACCGCCACACAG
GACACAAGTCTGCAGGATGGCTGTCTGATCTATAACGTGAAGATTCGCGGGGTCAACTTTCCCAGTAAT
GGACCTGTGATGCAGAAGAAAACCCTGGGATGGGAGGCTTCAACTGAAACCCTGTACCCAGCAGACGG
AGGACTGGAGGGACGAGCAGATATGGCTCTGAAACTGGTGGGCGGGGGACACCTGATCTGCAACCTG
AAGACTACCTATCGGTCCAAGAAACCTGCTAAGAATCTGAAAATGCCAGGCGTGTACTATGTGGACCGG
AGACTGGAGAGAATTAAGGAAGCAGATAAAGAGACCTACGTGGAGCAGCACGAAGTGGCTGTCGCAC
GATATTGTGACCTGCCTTCTAAACTGGGCCATCGGGGCGGGTCTATGGTGAGTAAGGGCGAGGAACTG
TTCACAGGGGTGGTCCCAATCCTGGTGGAACTGGACGGCGATGTCAATGGGCACAAGTTCAGCGTGTCC
GGAGAGGGAGAAGGGGACGCAACCTTTGGAGGCAGCGCCGACCTGGAAGATAATATGGAGACACTGA
ACGATAATCTGAAAGTGATCGAGAAAGCCGACAACGCCGCTCAGGTCAAGGATGCTCTGACTAAAATG
AGGGCAGCCGCTCTGGATGCACAGAAAGCCACCCCCCCTAAGCTGGAAGACAAATCACCTGATAGCCCA
GAGATGAAGGACTTCCGCCACGGATTTGATATCCTGGTCGGCCAGATTGACGATGCTCTGAAGCTGGCA

AATGAAGGCAAGGTGAAAGAGGCACAGGCAGCCGCTGAGCAGCTGAAAACAACTAGGAACGCCTACC
ATCAGAAGTATCGCGGGGGAAAGCTGACACTGAAATTCATCTGCACCACAGGCAAGCTGCCCGTGCCCT
GGCCAACTCTGGTCACTACCCTGGGATACGGCGTGCAGTGTTTTTCCCGCTATCCAGACCACATGAAGCA
GCATGATTTCTTTAAATCTGCCATGCCCGAAGGCTACGTGCAGGAGAGAACCATCTTCTTTAAGGACGAT
GGAAACTATAAAACAAGGGCTGAAGTGAAGTTCGAGGGAGACACTCTGGTCAACCGCATCGAACTGAA
GGGCATTGACTTTAAAGAGGATGGAAATATTCTGGGCCACAAGCTGGAATACAACTATAATAGCCATAA
CGTGTACATCATGGCCGATAAGCAGAAAAACGGCATTAAGGTCAATTTCAAAATCCGGCACAATATTGA
GGACGGGAGCGTGCAGCTGGCCGATCATTACCAGCAGAACACCCCAATCGGGGACGGACCAGTGCTGC
TGCCCGATAATCACTATCTGTCCACACAGTCTGCCCTGAGTAAGGACCCTAACGAAAAAGAGATCACAT
GGTGCTGCTGGAGTTTGTCACCGCAGCCGGGATTACACTGGGAATGGACGAGCTGTACAAGTGA

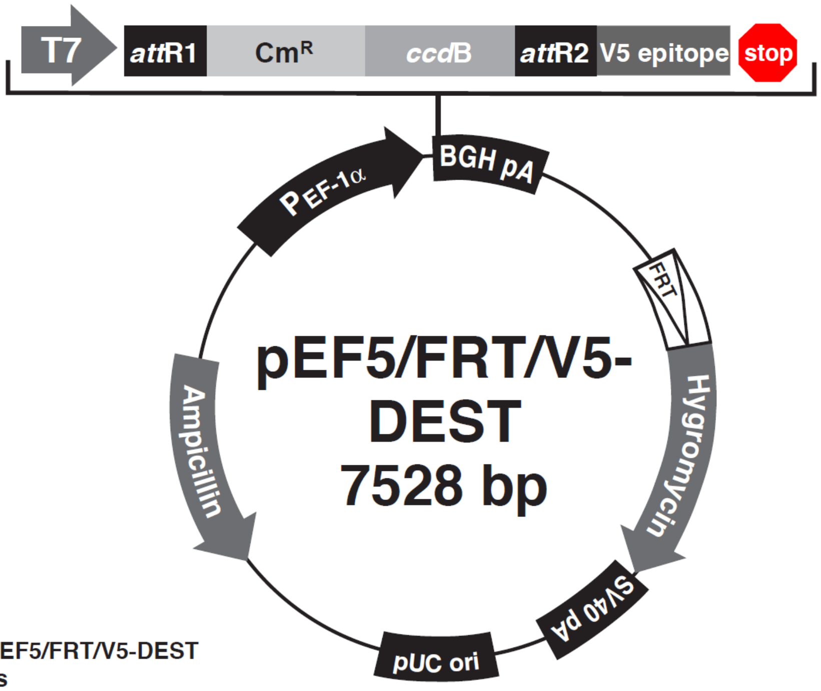

Comments for pEF5/FRT/V5-DEST
7528 nucleotides

EF-1α promoter: bases 348-1531
T7 promoter/priming site: bases 1548-1567
*att*R1 recombination site: bases 1645-1769
Chloramphenicol resistance gene: bases 1878-2537
*ccd*B gene: bases 2879-3184
*att*R2 recombination site: bases 3225-3349
V5 epitope: bases 3402-3443
BGH reverse priming site: bases 3481-3498
BGH polyadenylation signal: bases 3487-3711
FRT site: bases 3994-4041
Hygromycin resistance gene (no ATG): bases 4049-5069
SV40 early polyadenylation signal: bases 5201-5331
pUC origin: bases 5714-6387
bla promoter: bases 7393-7491 (complementary strand)
Ampicillin (*bla*) resistance gene: bases 6532-7392 (complementary strand)

Expression Clone pEF5FRTV5-DEST-hHS1

CTTGTACAAAGTGGTTGATATCCAGCACAGTGGCGGCCGCTCGAGTCTAGAGGGCCCGCGGTTCGAAG
GTAAGCCTATCCCTAACCCTCTCCTCGGTCTCGATTCTACGCGTACCGGTTAGTAATGAGTTTAAACCCGC
TGATCAGCCTCGACTGTGCCTTCTAGTTGCCAGCCATCTGTTGTTTGCCCCTCCCCCGTGCCTTCCTTGAC
CCTGGAAGGTGCCACTCCCACTGTCCTTTCCTAATAAAATGAGGAAATTGCATCGCATTGTCTGAGTAGG
TGTCATTCTATTCTGGGGGGTGGGGTGGGGCAGGACAGCAAGGGGGAGGATTGGGAAGACAATAGCA
GGCATGCTGGGGATGCGGTGGGCTCTATGGCTTCTGAGGCGGAAAGAACCAGCTGGGGCTCTAGGGG
GTATCCCCACGCGCCCTGTAGCGGCGCATTAAGCGCGGCGGGTGTGGTGGTTACGCGCAGCGTGACCG
CTACACTTGCCAGCGCCCTAGCGCCCGCTCCTTTCGCTTTCTTCCCTTCCTTTCTCGCCACGTTCGCCGGCT
TTCCCCGTCAAGCTCTAAATCGGGGGTCCCTTTAGGGTTCCGATTTAGTGCTTTACGGCACCTCGACCCC
AAAAAACTTGATTAGGGTGATGGTTCACGTACCTAGAAGTTCCTATTCCGAAGTTCCTATTCTCTAGAAA
GTATAGGAACTTCCTTGGCCAAAAAGCCTGAACTCACCGCGACGTCTGTCGAGAAGTTTCTGATCGAAA
AGTTCGACAGCGTCTCCGACCTGATGCAGCTCTCGGAGGGCGAAGAATCTCGTGCTTTCAGCTTCGATG
TAGGAGGGCGTGGATATGTCCTGCGGGTAAATAGCTGCGCCGATGGTTTCTACAAAGATCGTTATGTTT
ATCGGCACTTTGCATCGGCCGCGCTCCCGATTCCGGAAGTGCTTGACATTGGGGAATTCAGCGAGAGCC
TGACCTATTGCATCTCCCGCCGTGCACAGGGTGTCACGTTGCAAGACCTGCCTGAAACCGAACTGCCCGC
TGTTCTGCAGCCGGTCGCGGAGGCCATGGATGCGATCGCTGCGGCCGATCTTAGCCAGACGAGCGGGT
TCGGCCCATTCGGACCGCAAGGAATCGGTCAATACACTACATGGCGTGATTTCATATGCGCGATTGCTG
ATCCCCATGTGTATCACTGGCAAACTGTGATGGACGACACCGTCAGTGCGTCCGTCGCGCAGGCTCTCG
ATGAGCTGATGCTTTGGGCCGAGGACTGCCCCGAAGTCCGGCACCTCGTGCACGCGGATTTCGGCTCCA
ACAATGTCCTGACGGACAATGGCCGCATAACAGCGGTCATTGACTGGAGCGAGGCGATGTTCGGGGAT
TCCCAATACGAGGTCGCCAACATCTTCTTCTGGAGGCCGTGGTTGGCTTGTATGGAGCAGCAGACGCGC
TACTTCGAGCGGAGGCATCCGGAGCTTGCAGGATCGCCGCGGCTCCGGGCGTATATGCTCCGCATTGGT
CTTGACCAACTCTATCAGAGCTTGGTTGACGGCAATTTCGATGATGCAGCTTGGGCGCAGGGTCGATGC
GACGCAATCGTCCGATCCGGAGCCGGGACTGTCGGGCGTACACAAATCGCCCGCAGAAGCGCGGCCGT
CTGGACCGATGGCTGTGTAGAAGTACTCGCCGATAGTGGAAACCGACGCCCCAGCACTCGTCCGAGGG
CAAAGGAATAGCACGTACTACGAGATTTCGATTCCACCGCCGCCTTCTATGAAAGGTTGGGCTTCGGAA
TCGTTTTCCGGGACGCCGGCTGGATGATCCTCCAGCGCGGGGATCTCATGCTGGAGTTCTTCGCCCACCC
CAACTTGTTTATTGCAGCTTATAATGGTTACAAATAAAGCAATAGCATCACAAATTTCACAAATAAAGCA
TTTTTTTCACTGCATTCTAGTTGTGGTTTGTCCAAACTCATCAATGTATCTTATCATGTCTGTATACCGTCG
ACCTCTAGCTAGAGCTTGGCGTAATCATGGTCATAGCTGTTTCCTGTGTGAAATTGTTATCCGCTCACAAT
TCCACACAACATACGAGCCGGAAGCATAAAGTGTAAAGCCTGGGGTGCCTAATGAGTGAGCTAACTCAC
ATTAATTGCGTTGCGCTCACTGCCCGCTTTCCAGTCGGGAAACCTGTCGTGCCAGCTGCATTAATGAATC
GGCCAACGCGCGGGGAGAGGCGGTTTGCGTATTGGGCGCTCTTCCGCTTCCTCGCTCACTGACTCGCTG
CGCTCGGTCGTTCGGCTGCGGCGAGCGGTATCAGCTCACTCAAAGGCGGTAATACGGTTATCCACAGAA
TCAGGGGATAACGCAGGAAAGAACATGTGAGCAAAAGGCCAGCAAAAGGCCAGGAACCGTAAAAAGG
CCGCGTTGCTGGCGTTTTTCCATAGGCTCCGCCCCCCTGACGAGCATCACAAAAATCGACGCTCAAGTCA
GAGGTGGCGAAACCCGACAGGACTATAAAGATACCAGGCGTTTCCCCCTGGAAGCTCCCTCGTGCGCTC
TCCTGTTCCGACCCTGCCGCTTACCGGATACCTGTCCGCCTTTCTCCCTTCGGGAAGCGTGGCGCTTTCTC
ATAGCTCACGCTGTAGGTATCTCAGTTCGGTGTAGGTCGTTCGCTCCAAGCTGGGCTGTGTGCACGAAC

CCCCCGTTCAGCCCGACCGCTGCGCCTTATCCGGTAACTATCGTCTTGAGTCCAACCCGGTAAGACACGA
CTTATCGCCACTGGCAGCAGCCACTGGTAACAGGATTAGCAGAGCGAGGTATGTAGGCGGTGCTACAG
AGTTCTTGAAGTGGTGGCCTAACTACGGCTACACTAGAAGGACAGTATTTGGTATCTGCGCTCTGCTGA
AGCCAGTTACCTTCGGAAAAAGAGTTGGTAGCTCTTGATCCGGCAAACAAACCACCGCTGGTAGCGGTG
GTTTTTTTGTTTGCAAGCAGCAGATTACGCGCAGAAAAAAAGGATCTCAAGAAGATCCTTTGATCTTTTC
TACGGGGTCTGACGCTCAGTGGAACGAAAACTCACGTTAAGGGATTTTGGTCATGAGATTATCAAAAAG
GATCTTCACCTAGATCCTTTTAAATTAAAAATGAAGTTTTAAATCAATCTAAAGTATATATGAGTAAACTT
GGTCTGACAGTTACCAATGCTTAATCAGTGAGGCACCTATCTCAGCGATCTGTCTATTTCGTTCATCCATA
GTTGCCTGACTCCCCGTCGTGTAGATAACTACGATACGGGAGGGCTTACCATCTGGCCCCAGTGCTGCA
ATGATACCGCGAGACCCACGCTCACCGGCTCCAGATTTATCAGCAATAAACCAGCCAGCCGGAAGGGCC
GAGCGCAGAAGTGGTCCTGCAACTTTATCCGCCTCCATCCAGTCTATTAATTGTTGCCGGGAAGCTAGAG
TAAGTAGTTCGCCAGTTAATAGTTTGCGCAACGTTGTTGCCATTGCTACAGGCATCGTGGTGTCACGCTC
GTCGTTTGGTATGGCTTCATTCAGCTCCGGTTCCCAACGATCAAGGCGAGTTACATGATCCCCCATGTTG
TGCAAAAAAGCGGTTAGCTCCTTCGGTCCTCCGATCGTTGTCAGAAGTAAGTTGGCCGCAGTGTTATCAC
TCATGGTTATGGCAGCACTGCATAATTCTCTTACTGTCATGCCATCCGTAAGATGCTTTTCTGTGACTGGT
GAGTACTCAACCAAGTCATTCTGAGAATAGTGTATGCGGCGACCGAGTTGCTCTTGCCCGGCGTCAATA
CGGGATAATACCGCGCCACATAGCAGAACTTTAAAAGTGCTCATCATTGGAAAACGTTCTTCGGGGCGA
AAACTCTCAAGGATCTTACCGCTGTTGAGATCCAGTTCGATGTAACCCACTCGTGCACCCAACTGATCTTC
AGCATCTTTTACTTTCACCAGCGTTTCTGGGTGAGCAAAAACAGGAAGGCAAAATGCCGCAAAAAAGGG
AATAAGGGCGACACGGAAATGTTGAATACTCATACTCTTCCTTTTTCAATATTATTGAAGCATTTATCAGG
GTTATTGTCTCATGAGCGGATACATATTTGAATGTATTTAGAAAAATAAACAAATAGGGGTTCCGCGCAC
ATTTCCCCGAAAAGTGCCACCTGACGTCGACGGATCGGGAGATCTCCCGATCCCCTATGGTGCACTCTCA
GTACAATCTGCTCTGATGCCGCATAGTTAAGCCAGTATCTGCTCCCTGCTTGTGTGTTGGAGGTCGCTGA
GTAGTGCGCGAGCAAAATTTAAGCTACAACAAGGCAAGGCTTGACCGACAATTGCATGAAGAATCTGCT
TAGGGTTAGGCGTTTTGCGCTGCTTCGCGATGTACGGGCCAGATATACGCGTTGACATTGATTATTGACT
AGGCTTTTGCAAAAAGCTTTGCAAAGATGGATAAAGTTTTAAACAGAGAGGAATCTTTGCAGCTAATGG
ACCTTCTAGGTCTTGAAAGGAGTGCCTCGTGAGGCTCCGGTGCCCGTCAGTGGGCAGAGCGCACATCGC
CCACAGTCCCCGAGAAGTTGGGGGGAGGGGTCGGCAATTGAACCGGTGCCTAGAGAAGGTGGCGCGG
GGTAAACTGGGAAAGTGATGTCGTGTACTGGCTCCGCCTTTTTCCCGAGGGTGGGGGAGAACCGTATAT
AAGTGCAGTAGTCGCCGTGAACGTTCTTTTTCGCAACGGGTTTGCCGCCAGAACACAGGTAAGTGCCGT
GTGTGGTTCCCGCGGGCCTGGCCTCTTTACGGGTTATGGCCCTTGCGTGCCTTGAATTACTTCCACCTGG
CTGCAGTACGTGATTCTTGATCCCGAGCTTCGGGTTGGAAGTGGGTGGGAGAGTTCGAGGCCTTGCGCT
TAAGGAGCCCCTTCGCCTCGTGCTTGAGTTGAGGCCTGGCCTGGGCGCTGGGGCCGCCGCGTGCGAAT
CTGGTGGCACCTTCGCGCCTGTCTCGCTGCTTTCGATAAGTCTCTAGCCATTTAAAATTTTTGATGACCTG
CTGCGACGCTTTTTTTCTGGCAAGATAGTCTTGTAAATGCGGGCCAAGATCTGCACACTGGTATTTCGGT
TTTTGGGGCCGCGGGCGGCGACGGGGCCCGTGCGTCCCAGCGCACATGTTCGGCGAGGCGGGGCCTG
CGAGCGCGGCCACCGAGAATCGGACGGGGGTAGTCTCAAGCTGGCCGGCCTGCTCTGGTGCCTGGCCT
CGCGCCGCCGTGTATCGCCCCGCCCTGGGCGGCAAGGCTGGCCCGGTCGGCACCAGTTGCGTGAGCGG
AAAGATGGCCGCTTCCCGGCCCTGCTGCAGGGAGCTCAAAATGGAGGACGCGGCGCTCGGGAGAGCG
GGCGGGTGAGTCACCCACACAAAGGAAAAGGGCCTTTCCGTCCTCAGCCGTCGCTTCATGTGACTCCAC
GGAGTACCGGGCGCCGTCCAGGCACCTCGATTAGTTCTCGAGCTTTTGGAGTACGTCGTCTTTAGGTTG

GGGGGAGGGGTTTTATGCGATGGAGTTTCCCCACACTGAGTGGGTGGAGACTGAAGTTAGGCCAGCTT
GGCACTTGATGTAATTCTCCTTGGAATTTGCCCTTTTTGAGTTTGGATCTTGGTTCATTCTCAAGCCTCAG
ACAGTGGTTCAAAGTTTTTTTCTTCCATTTCAGGTGTCGTGAGGAATTAGCTTGGTACTAATACGACTCAC
TATAGGGAGACCCAAGCTGGCTAGGTAAGCTTGGTACCGAGCTCGGATCCACTAGTCCAGTGTGGTGG
AATTCTGCAGATATCAACAAGTTTGTACAAAAAAGCAGGCTCCGCCACCATGCACATGGTCAGCGAGCT
GATCAAGGAAAACATGCACATGAAACTGTACATGGAGGGGACTGTGAACAATCACCATTTCAAATGCAC
CTCCGAGGGCGAAGGGAAGCCCTACGAGGGCACACAGACTATGAGGATCAAGGCAGTGGAGGGAGGA
CCACTGCCATTCGCCTTTGACATTCTGGCTACCTCATTCATGTACGGCAGCAAAACCTTCATCAATCACAC
TCAGGGGATTCCCGACTTCTTTAAGCAGTCTTTCCCTGAAGGCTTTACTTGGGAGCGAGTGACCACATAC
GAGGATGGAGGCGTCCTGACCGCCACACAGGACACAAGTCTGCAGGATGGCTGTCTGATCTATAACGT
GAAGATTCGCGGGGTCAACTTTCCCAGTAATGGACCTGTGATGCAGAAGAAAACCCTGGGATGGGAGG
CTTCAACTGAAACCCTGTACCCAGCAGACGGAGGACTGGAGGGACGAGCAGATATGGCTCTGAAACTG
GTGGGCGGGGGACACCTGATCTGCAACCTGAAGACTACCTATCGGTCCAAGAAACCTGCTAAGAATCTG
AAAATGCCAGGCGTGTACTATGTGGACCGGAGACTGGAGAGAATTAAGGAAGCAGATAAAGAGACCTA
CGTGGAGCAGCACGAAGTGGCTGTCGCACGATATTGTGACCTGCCTTCTAAACTGGGCCATCGGGGCG
GGTCTATGGTGAGTAAGGGCGAGGAACTGTTCACAGGGGTGGTCCCAATCCTGGTGGAACTGGACGGC
GATGTCAATGGGCACAAGTTCAGCGTGTCCGGAGAGGGAGAAGGGGACGCAACCTTTGGAGGCAGCG
CCGACCTGGAAGATAATATGGAGACACTGAACGATAATCTGAAAGTGATCGAGAAAGCCGACAACGCC
GCTCAGGTCAAGGATGCTCTGACTAAAATGAGGGCAGCCGCTCTGGATGCACAGAAAGCCACCCCCCCT
AAGCTGGAAGACAAATCACCTGATAGCCCAGAGATGAAGGACTTCCGCCACGGATTTGATATCCTGGTC
GGCCAGATTGACGATGCTCTGAAGCTGGCAAATGAAGGCAAGGTGAAAGAGGCACAGGCAGCCGCTG
AGCAGCTGAAAACAACTAGGAACGCCTACCATCAGAAGTATCGCGGGGGAAAGCTGACACTGAAATTC
ATCTGCACCACAGGCAAGCTGCCCGTGCCCTGGCCAACTCTGGTCACTACCCTGGGATACGGCGTGCAG
TGTTTTTCCCGCTATCCAGACCACATGAAGCAGCATGATTTCTTTAAATCTGCCATGCCCGAAGGCTACGT
GCAGGAGAGAACCATCTTCTTTAAGGACGATGGAAACTATAAAACAAGGGCTGAAGTGAAGTTCGAGG
GAGACACTCTGGTCAACCGCATCGAACTGAAGGGCATTGACTTTAAAGAGGATGGAAATATTCTGGGCC
ACAAGCTGGAATACAACTATAATAGCCATAACGTGTACATCATGGCCGATAAGCAGAAAAACGGCATTA
AGGTCAATTTCAAAATCCGGCACAATATTGAGGACGGGAGCGTGCAGCTGGCCGATCATTACCAGCAG
AACACCCCAATCGGGGACGGACCAGTGCTGCTGCCCGATAATCACTATCTGTCCACACAGTCTGCCCTGA
GTAAGGACCCTAACGAAAAAAGAGATCACATGGTGCTGCTGGAGTTTGTCACCGCAGCCGGGATTACA
CTGGGAATGGACGAGCTGTACAAGTGAGACCCAGCTTT

Expression Clone pEF5FRTV5-DESTNLS-hHS1

CTTGTACAAAGTGGTTGATATCCAGCACAGTGGCGGCCGCTCGAGTCTAGAGGGCCCGCGGTTCGAAG
GTAAGCCTATCCCTAACCCTCTCCTCGGTCTCGATTCTACGCGTACCGGTTAGTAATGAGTTTAAACCCGC
TGATCAGCCTCGACTGTGCCTTCTAGTTGCCAGCCATCTGTTGTTTGCCCCTCCCCCGTGCCTTCCTTGAC
CCTGGAAGGTGCCACTCCCACTGTCCTTTCCTAATAAAATGAGGAAATTGCATCGCATTGTCTGAGTAGG
TGTCATTCTATTCTGGGGGGTGGGGTGGGGCAGGACAGCAAGGGGGAGGATTGGGAAGACAATAGCA
GGCATGCTGGGGATGCGGTGGGCTCTATGGCTTCTGAGGCGGAAAGAACCAGCTGGGGCTCTAGGGG

GTATCCCCACGCGCCCTGTAGCGGCGCATTAAGCGCGGCGGGTGTGGTGGTTACGCGCAGCGTGACCG
CTACACTTGCCAGCGCCCTAGCGCCCGCTCCTTTCGCTTTCTTCCCTTCCTTTCTCGCCACGTTCGCCGGCT
TTCCCCGTCAAGCTCTAAATCGGGGGTCCCTTTAGGGTTCCGATTTAGTGCTTTACGGCACCTCGACCCC
AAAAAACTTGATTAGGGTGATGGTTCACGTACCTAGAAGTTCCTATTCCGAAGTTCCTATTCTCTAGAAA
GTATAGGAACTTCCTTGGCCAAAAAGCCTGAACTCACCGCGACGTCTGTCGAGAAGTTTCTGATCGAAA
AGTTCGACAGCGTCTCCGACCTGATGCAGCTCTCGGAGGGCGAAGAATCTCGTGCTTTCAGCTTCGATG
TAGGAGGGCGTGGATATGTCCTGCGGGTAAATAGCTGCGCCGATGGTTTCTACAAAGATCGTTATGTTT
ATCGGCACTTTGCATCGGCCGCGCTCCCGATTCCGGAAGTGCTTGACATTGGGGAATTCAGCGAGAGCC
TGACCTATTGCATCTCCCGCCGTGCACAGGGTGTCACGTTGCAAGACCTGCCTGAAACCGAACTGCCCGC
TGTTCTGCAGCCGGTCGCGGAGGCCATGGATGCGATCGCTGCGGCCGATCTTAGCCAGACGAGCGGGT
TCGGCCCATTCGGACCGCAAGGAATCGGTCAATACACTACATGGCGTGATTTCATATGCGCGATTGCTG
ATCCCCATGTGTATCACTGGCAAACTGTGATGGACGACACCGTCAGTGCGTCCGTCGCGCAGGCTCTCG
ATGAGCTGATGCTTTGGGCCGAGGACTGCCCCGAAGTCCGGCACCTCGTGCACGCGGATTTCGGCTCCA
ACAATGTCCTGACGGACAATGGCCGCATAACAGCGGTCATTGACTGGAGCGAGGCGATGTTCGGGGAT
TCCCAATACGAGGTCGCCAACATCTTCTTCTGGAGGCCGTGGTTGGCTTGTATGGAGCAGCAGACGCGC
TACTTCGAGCGGAGGCATCCGGAGCTTGCAGGATCGCCGCGGCTCCGGGCGTATATGCTCCGCATTGGT
CTTGACCAACTCTATCAGAGCTTGGTTGACGGCAATTTCGATGATGCAGCTTGGGCGCAGGGTCGATGC
GACGCAATCGTCCGATCCGGAGCCGGGACTGTCGGGCGTACACAAATCGCCCGCAGAAGCGCGGCCGT
CTGGACCGATGGCTGTGTAGAAGTACTCGCCGATAGTGGAAACCGACGCCCCAGCACTCGTCCGAGGG
CAAAGGAATAGCACGTACTACGAGATTTCGATTCCACCGCCGCCTTCTATGAAAGGTTGGGCTTCGGAA
TCGTTTTCCGGGACGCCGGCTGGATGATCCTCCAGCGCGGGGATCTCATGCTGGAGTTCTTCGCCCACCC
CAACTTGTTTATTGCAGCTTATAATGGTTACAAATAAAGCAATAGCATCACAAATTTCACAAATAAAGCA
TTTTTTTCACTGCATTCTAGTTGTGGTTTGTCCAAACTCATCAATGTATCTTATCATGTCTGTATACCGTCG
ACCTCTAGCTAGAGCTTGGCGTAATCATGGTCATAGCTGTTTCCTGTGTGAAATTGTTATCCGCTCACAAT
TCCACACAACATACGAGCCGGAAGCATAAAGTGTAAAGCCTGGGGTGCCTAATGAGTGAGCTAACTCAC
ATTAATTGCGTTGCGCTCACTGCCCGCTTTCCAGTCGGGAAACCTGTCGTGCCAGCTGCATTAATGAATC
GGCCAACGCGCGGGGAGAGGCGGTTTGCGTATTGGGCGCTCTTCCGCTTCCTCGCTCACTGACTCGCTG
CGCTCGGTCGTTCGGCTGCGGCGAGCGGTATCAGCTCACTCAAAGGCGGTAATACGGTTATCCACAGAA
TCAGGGGATAACGCAGGAAAGAACATGTGAGCAAAAGGCCAGCAAAAGGCCAGGAACCGTAAAAAGG
CCGCGTTGCTGGCGTTTTTCCATAGGCTCCGCCCCCCTGACGAGCATCACAAAAATCGACGCTCAAGTCA
GAGGTGGCGAAACCCGACAGGACTATAAAGATACCAGGCGTTTCCCCCTGGAAGCTCCCTCGTGCGCTC
TCCTGTTCCGACCCTGCCGCTTACCGGATACCTGTCCGCCTTTCTCCCTTCGGGAAGCGTGGCGCTTTCTC
ATAGCTCACGCTGTAGGTATCTCAGTTCGGTGTAGGTCGTTCGCTCCAAGCTGGGCTGTGTGCACGAAC
CCCCCGTTCAGCCCGACCGCTGCGCCTTATCCGGTAACTATCGTCTTGAGTCCAACCCGGTAAGACACGA
CTTATCGCCACTGGCAGCAGCCACTGGTAACAGGATTAGCAGAGCGAGGTATGTAGGCGGTGCTACAG
AGTTCTTGAAGTGGTGGCCTAACTACGGCTACACTAGAAGGACAGTATTTGGTATCTGCGCTCTGCTGA
AGCCAGTTACCTTCGGAAAAAGAGTTGGTAGCTCTTGATCCGGCAAACAAACCACCGCTGGTAGCGGTG
GTTTTTTTGTTTGCAAGCAGCAGATTACGCGCAGAAAAAAAGGATCTCAAGAAGATCCTTTGATCTTTTC
TACGGGGTCTGACGCTCAGTGGAACGAAAACTCACGTTAAGGGATTTTGGTCATGAGATTATCAAAAAG
GATCTTCACCTAGATCCTTTTAAATTAAAAATGAAGTTTTAAATCAATCTAAAGTATATATGAGTAAACTT
GGTCTGACAGTTACCAATGCTTAATCAGTGAGGCACCTATCTCAGCGATCTGTCTATTTCGTTCATCCATA

GTTGCCTGACTCCCCGTCGTGTAGATAACTACGATACGGGAGGGCTTACCATCTGGCCCCAGTGCTGCA
ATGATACCGCGAGACCCACGCTCACCGGCTCCAGATTTATCAGCAATAAACCAGCCAGCCGGAAGGGCC
GAGCGCAGAAGTGGTCCTGCAACTTTATCCGCCTCCATCCAGTCTATTAATTGTTGCCGGGAAGCTAGAG
TAAGTAGTTCGCCAGTTAATAGTTTGCGCAACGTTGTTGCCATTGCTACAGGCATCGTGGTGTCACGCTC
GTCGTTTGGTATGGCTTCATTCAGCTCCGGTTCCCAACGATCAAGGCGAGTTACATGATCCCCCATGTTG
TGCAAAAAAGCGGTTAGCTCCTTCGGTCCTCCGATCGTTGTCAGAAGTAAGTTGGCCGCAGTGTTATCAC
TCATGGTTATGGCAGCACTGCATAATTCTCTTACTGTCATGCCATCCGTAAGATGCTTTTCTGTGACTGGT
GAGTACTCAACCAAGTCATTCTGAGAATAGTGTATGCGGCGACCGAGTTGCTCTTGCCCGGCGTCAATA
CGGGATAATACCGCGCCACATAGCAGAACTTTAAAAGTGCTCATCATTGGAAAACGTTCTTCGGGGCGA
AAACTCTCAAGGATCTTACCGCTGTTGAGATCCAGTTCGATGTAACCCACTCGTGCACCCAACTGATCTTC
AGCATCTTTTACTTTCACCAGCGTTTCTGGGTGAGCAAAAACAGGAAGGCAAAATGCCGCAAAAAAGGG
AATAAGGGCGACACGGAAATGTTGAATACTCATACTCTTCCTTTTTCAATATTATTGAAGCATTTATCAGG
GTTATTGTCTCATGAGCGGATACATATTTGAATGTATTTAGAAAAATAAACAAATAGGGGTTCCGCGCAC
ATTTCCCCGAAAAGTGCCACCTGACGTCGACGGATCGGGAGATCTCCCGATCCCCTATGGTGCACTCTCA
GTACAATCTGCTCTGATGCCGCATAGTTAAGCCAGTATCTGCTCCCTGCTTGTGTGTTGGAGGTCGCTGA
GTAGTGCGCGAGCAAAATTTAAGCTACAACAAGGCAAGGCTTGACCGACAATTGCATGAAGAATCTGCT
TAGGGTTAGGCGTTTTGCGCTGCTTCGCGATGTACGGGCCAGATATACGCGTTGACATTGATTATTGACT
AGGCTTTTGCAAAAAGCTTTGCAAAGATGGATAAAGTTTTAAACAGAGAGGAATCTTTGCAGCTAATGG
ACCTTCTAGGTCTTGAAAGGAGTGCCTCGTGAGGCTCCGGTGCCCGTCAGTGGGCAGAGCGCACATCGC
CCACAGTCCCCGAGAAGTTGGGGGGAGGGGTCGGCAATTGAACCGGTGCCTAGAGAAGGTGGCGCGG
GGTAAACTGGGAAAGTGATGTCGTGTACTGGCTCCGCCTTTTTCCCGAGGGTGGGGGAGAACCGTATAT
AAGTGCAGTAGTCGCCGTGAACGTTCTTTTTCGCAACGGGTTTGCCGCCAGAACACAGGTAAGTGCCGT
GTGTGGTTCCCGCGGGCCTGGCCTCTTTACGGGTTATGGCCCTTGCGTGCCTTGAATTACTTCCACCTGG
CTGCAGTACGTGATTCTTGATCCCGAGCTTCGGGTTGGAAGTGGGTGGGAGAGTTCGAGGCCTTGCGCT
TAAGGAGCCCCTTCGCCTCGTGCTTGAGTTGAGGCCTGGCCTGGGCGCTGGGGCCGCCGCGTGCGAAT
CTGGTGGCACCTTCGCGCCTGTCTCGCTGCTTTCGATAAGTCTCTAGCCATTTAAAATTTTTGATGACCTG
CTGCGACGCTTTTTTTCTGGCAAGATAGTCTTGTAAATGCGGGCCAAGATCTGCACACTGGTATTTCGGT
TTTTGGGGCCGCGGGCGGCGACGGGGCCCGTGCGTCCCAGCGCACATGTTCGGCGAGGCGGGGCCTG
CGAGCGCGGCCACCGAGAATCGGACGGGGGTAGTCTCAAGCTGGCCGGCCTGCTCTGGTGCCTGGCCT
CGCGCCGCCGTGTATCGCCCCGCCCTGGGCGGCAAGGCTGGCCCGGTCGGCACCAGTTGCGTGAGCGG
AAAGATGGCCGCTTCCCGGCCCTGCTGCAGGGAGCTCAAAATGGAGGACGCGGCGCTCGGGAGAGCG
GGCGGGTGAGTCACCCACACAAAGGAAAAGGGCCTTTCCGTCCTCAGCCGTCGCTTCATGTGACTCCAC
GGAGTACCGGGCGCCGTCCAGGCACCTCGATTAGTTCTCGAGCTTTTGGAGTACGTCGTCTTTAGGTTG
GGGGGAGGGGTTTTATGCGATGGAGTTTCCCCACACTGAGTGGGTGGAGACTGAAGTTAGGCCAGCTT
GGCACTTGATGTAATTCTCCTTGGAATTTGCCCTTTTTGAGTTTGGATCTTGGTTCATTCTCAAGCCTCAG
ACAGTGGTTCAAAGTTTTTTTCTTCCATTTCAGGTGTCGTGAGGAATTAGCTTGGTACTAATACGACTCAC
TATAGGGAGACCCAAGCTGGCTAGGTAAGCTTGGTACCGAGCTCGGATCCACTAGTCCAGTGTGGTGG
AATTCTGCAGATATCAACAAGTTTGTACAAAAAAGCAGGCTCCGCCACCATGGATCCAAAAAAGAAGAG
AAAGGTAGATCCAAAAAAGAAGAGAAAGGTAATGCACATGGTCAGCGAGCTGATCAAGGAAAACATG
CACATGAAACTGTACATGGAGGGGACTGTGAACAATCACCATTTCAAATGCACCTCCGAGGGCGAAGG
GAAGCCCTACGAGGGCACACAGACTATGAGGATCAAGGCAGTGGAGGGAGGACCACTGCCATTCGCCT

TTGACATTCTGGCTACCTCATTCATGTACGGCAGCAAAACCTTCATCAATCACACTCAGGGGATTCCCGA
CTTCTTTAAGCAGTCTTTCCCTGAAGGCTTTACTTGGGAGCGAGTGACCACATACGAGGATGGAGGCGT
CCTGACCGCCACACAGGACACAAGTCTGCAGGATGGCTGTCTGATCTATAACGTGAAGATTCGCGGGGT
CAACTTTCCCAGTAATGGACCTGTGATGCAGAAGAAAACCCTGGGATGGGAGGCTTCAACTGAAACCCT
GTACCCAGCAGACGGAGGACTGGAGGGACGAGCAGATATGGCTCTGAAACTGGTGGGCGGGGGACAC
CTGATCTGCAACCTGAAGACTACCTATCGGTCCAAGAAACCTGCTAAGAATCTGAAAATGCCAGGCGTG
TACTATGTGGACCGGAGACTGGAGAGAATTAAGGAAGCAGATAAAGAGACCTACGTGGAGCAGCACG
AAGTGGCTGTCGCACGATATTGTGACCTGCCTTCTAAACTGGGCCATCGGGGCGGGTCTATGGTGAGTA
AGGGCGAGGAACTGTTCACAGGGGTGGTCCCAATCCTGGTGGAACTGGACGGCGATGTCAATGGGCAC
AAGTTCAGCGTGTCCGGAGAGGGAGAAGGGGACGCAACCTTTGGAGGCAGCGCCGACCTGGAAGATA
ATATGGAGACACTGAACGATAATCTGAAAGTGATCGAGAAAGCCGACAACGCCGCTCAGGTCAAGGAT
GCTCTGACTAAAATGAGGGCAGCCGCTCTGGATGCACAGAAAGCCACCCCCCCTAAGCTGGAAGACAA
ATCACCTGATAGCCCAGAGATGAAGGACTTCCGCCACGGATTTGATATCCTGGTCGGCCAGATTGACGA
TGCTCTGAAGCTGGCAAATGAAGGCAAGGTGAAAGAGGCACAGGCAGCCGCTGAGCAGCTGAAAACA
ACTAGGAACGCCTACCATCAGAAGTATCGCGGGGGAAAGCTGACACTGAAATTCATCTGCACCACAGGC
AAGCTGCCCGTGCCCTGGCCAACTCTGGTCACTACCCTGGGATACGGCGTGCAGTGTTTTTCCCGCTATC
CAGACCACATGAAGCAGCATGATTTCTTTAAATCTGCCATGCCCGAAGGCTACGTGCAGGAGAGAACCA
TCTTCTTTAAGGACGATGGAAACTATAAAACAAGGGCTGAAGTGAAGTTCGAGGGAGACACTCTGGTCA
ACCGCATCGAACTGAAGGGCATTGACTTTAAAGAGGATGGAAATATTCTGGGCCACAAGCTGGAATACA
ACTATAATAGCCATAACGTGTACATCATGGCCGATAAGCAGAAAAACGGCATTAAGGTCAATTTCAAAA
TCCGGCACAATATTGAGGACGGGAGCGTGCAGCTGGCCGATCATTACCAGCAGAACACCCCAATCGGG
GACGGACCAGTGCTGCTGCCCGATAATCACTATCTGTCCACACAGTCTGCCCTGAGTAAGGACCCTAAC
GAAAAAAGAGATCACATGGTGCTGCTGGAGTTTGTCACCGCAGCCGGGATTACACTGGGAATGGACGA
GCTGTACAAGTGAGACCCAGCTTT

Expression ClonepEF5FRTV5-DESTmito-hHS1

CTTGTACAAAGTGGTTGATATCCAGCACAGTGGCGGCCGCTCGAGTCTAGAGGGCCCGCGGTTCGAAG
GTAAGCCTATCCCTAACCCTCTCCTCGGTCTCGATTCTACGCGTACCGGTTAGTAATGAGTTTAAACCCGC
TGATCAGCCTCGACTGTGCCTTCTAGTTGCCAGCCATCTGTTGTTTGCCCCTCCCCCGTGCCTTCCTTGAC
CCTGGAAGGTGCCACTCCCACTGTCCTTTCCTAATAAAATGAGGAAATTGCATCGCATTGTCTGAGTAGG
TGTCATTCTATTCTGGGGGGTGGGGTGGGGCAGGACAGCAAGGGGGAGGATTGGGAAGACAATAGCA
GGCATGCTGGGGATGCGGTGGGCTCTATGGCTTCTGAGGCGGAAAGAACCAGCTGGGGCTCTAGGGG
GTATCCCCACGCGCCCTGTAGCGGCGCATTAAGCGCGGCGGGTGTGGTGGTTACGCGCAGCGTGACCG
CTACACTTGCCAGCGCCCTAGCGCCCGCTCCTTTCGCTTTCTTCCCTTCCTTTCTCGCCACGTTCGCCGGCT
TTCCCCGTCAAGCTCTAAATCGGGGGTCCCTTTAGGGTTCCGATTTAGTGCTTTACGGCACCTCGACCCC
AAAAAACTTGATTAGGGTGATGGTTCACGTACCTAGAAGTTCCTATTCCGAAGTTCCTATTCTCTAGAAA
GTATAGGAACTTCCTTGGCCAAAAAGCCTGAACTCACCGCGACGTCTGTCGAGAAGTTTCTGATCGAAA
AGTTCGACAGCGTCTCCGACCTGATGCAGCTCTCGGAGGGCGAAGAATCTCGTGCTTTCAGCTTCGATG
TAGGAGGGCGTGGATATGTCCTGCGGGTAAATAGCTGCGCCGATGGTTTCTACAAAGATCGTTATGTTT

ATCGGCACTTTGCATCGGCCGCGCTCCCGATTCCGGAAGTGCTTGACATTGGGGAATTCAGCGAGAGCC
TGACCTATTGCATCTCCCGCCGTGCACAGGGTGTCACGTTGCAAGACCTGCCTGAAACCGAACTGCCCGC
TGTTCTGCAGCCGGTCGCGGAGGCCATGGATGCGATCGCTGCGGCCGATCTTAGCCAGACGAGCGGGT
TCGGCCCATTCGGACCGCAAGGAATCGGTCAATACACTACATGGCGTGATTTCATATGCGCGATTGCTG
ATCCCCATGTGTATCACTGGCAAACTGTGATGGACGACACCGTCAGTGCGTCCGTCGCGCAGGCTCTCG
ATGAGCTGATGCTTTGGGCCGAGGACTGCCCCGAAGTCCGGCACCTCGTGCACGCGGATTTCGGCTCCA
ACAATGTCCTGACGGACAATGGCCGCATAACAGCGGTCATTGACTGGAGCGAGGCGATGTTCGGGGAT
TCCCAATACGAGGTCGCCAACATCTTCTTCTGGAGGCCGTGGTTGGCTTGTATGGAGCAGCAGACGCGC
TACTTCGAGCGGAGGCATCCGGAGCTTGCAGGATCGCCGCGGCTCCGGGCGTATATGCTCCGCATTGGT
CTTGACCAACTCTATCAGAGCTTGGTTGACGGCAATTTCGATGATGCAGCTTGGGCGCAGGGTCGATGC
GACGCAATCGTCCGATCCGGAGCCGGGACTGTCGGGCGTACACAAATCGCCCGCAGAAGCGCGGCCGT
CTGGACCGATGGCTGTGTAGAAGTACTCGCCGATAGTGGAAACCGACGCCCCAGCACTCGTCCGAGGG
CAAAGGAATAGCACGTACTACGAGATTTCGATTCCACCGCCGCCTTCTATGAAAGGTTGGGCTTCGGAA
TCGTTTTCCGGGACGCCGGCTGGATGATCCTCCAGCGCGGGGATCTCATGCTGGAGTTCTTCGCCCACCC
CAACTTGTTTATTGCAGCTTATAATGGTTACAAATAAAGCAATAGCATCACAAATTTCACAAATAAAGCA
TTTTTTTCACTGCATTCTAGTTGTGGTTTGTCCAAACTCATCAATGTATCTTATCATGTCTGTATACCGTCG
ACCTCTAGCTAGAGCTTGGCGTAATCATGGTCATAGCTGTTTCCTGTGTGAAATTGTTATCCGCTCACAAT
TCCACACAACATACGAGCCGGAAGCATAAAGTGTAAAGCCTGGGGTGCCTAATGAGTGAGCTAACTCAC
ATTAATTGCGTTGCGCTCACTGCCCGCTTTCCAGTCGGGAAACCTGTCGTGCCAGCTGCATTAATGAATC
GGCCAACGCGCGGGGAGAGGCGGTTTGCGTATTGGGCGCTCTTCCGCTTCCTCGCTCACTGACTCGCTG
CGCTCGGTCGTTCGGCTGCGGCGAGCGGTATCAGCTCACTCAAAGGCGGTAATACGGTTATCCACAGAA
TCAGGGGATAACGCAGGAAAGAACATGTGAGCAAAAGGCCAGCAAAAGGCCAGGAACCGTAAAAAGG
CCGCGTTGCTGGCGTTTTTCCATAGGCTCCGCCCCCCTGACGAGCATCACAAAAATCGACGCTCAAGTCA
GAGGTGGCGAAACCCGACAGGACTATAAAGATACCAGGCGTTTCCCCCTGGAAGCTCCCTCGTGCGCTC
TCCTGTTCCGACCCTGCCGCTTACCGGATACCTGTCCGCCTTTCTCCCTTCGGGAAGCGTGGCGCTTTCTC
ATAGCTCACGCTGTAGGTATCTCAGTTCGGTGTAGGTCGTTCGCTCCAAGCTGGGCTGTGTGCACGAAC
CCCCCGTTCAGCCCGACCGCTGCGCCTTATCCGGTAACTATCGTCTTGAGTCCAACCCGGTAAGACACGA
CTTATCGCCACTGGCAGCAGCCACTGGTAACAGGATTAGCAGAGCGAGGTATGTAGGCGGTGCTACAG
AGTTCTTGAAGTGGTGGCCTAACTACGGCTACACTAGAAGGACAGTATTTGGTATCTGCGCTCTGCTGA
AGCCAGTTACCTTCGGAAAAAGAGTTGGTAGCTCTTGATCCGGCAAACAAACCACCGCTGGTAGCGGTG
GTTTTTTTGTTTGCAAGCAGCAGATTACGCGCAGAAAAAAAGGATCTCAAGAAGATCCTTTGATCTTTTC
TACGGGGTCTGACGCTCAGTGGAACGAAAACTCACGTTAAGGGATTTTGGTCATGAGATTATCAAAAAG
GATCTTCACCTAGATCCTTTTAAATTAAAAATGAAGTTTTAAATCAATCTAAAGTATATATGAGTAAACTT
GGTCTGACAGTTACCAATGCTTAATCAGTGAGGCACCTATCTCAGCGATCTGTCTATTTCGTTCATCCATA
GTTGCCTGACTCCCCGTCGTGTAGATAACTACGATACGGGAGGGCTTACCATCTGGCCCCAGTGCTGCA
ATGATACCGCGAGACCCACGCTCACCGGCTCCAGATTTATCAGCAATAAACCAGCCAGCCGGAAGGGCC
GAGCGCAGAAGTGGTCCTGCAACTTTATCCGCCTCCATCCAGTCTATTAATTGTTGCCGGGAAGCTAGAG
TAAGTAGTTCGCCAGTTAATAGTTTGCGCAACGTTGTTGCCATTGCTACAGGCATCGTGGTGTCACGCTC
GTCGTTTGGTATGGCTTCATTCAGCTCCGGTTCCCAACGATCAAGGCGAGTTACATGATCCCCCATGTTG
TGCAAAAAAGCGGTTAGCTCCTTCGGTCCTCCGATCGTTGTCAGAAGTAAGTTGGCCGCAGTGTTATCAC
TCATGGTTATGGCAGCACTGCATAATTCTCTTACTGTCATGCCATCCGTAAGATGCTTTTCTGTGACTGGT

GAGTACTCAACCAAGTCATTCTGAGAATAGTGTATGCGGCGACCGAGTTGCTCTTGCCCGGCGTCAATA
CGGGATAATACCGCGCCACATAGCAGAACTTTAAAAGTGCTCATCATTGGAAAACGTTCTTCGGGGCGA
AAACTCTCAAGGATCTTACCGCTGTTGAGATCCAGTTCGATGTAACCCACTCGTGCACCCAACTGATCTTC
AGCATCTTTTACTTTCACCAGCGTTTCTGGGTGAGCAAAAACAGGAAGGCAAAATGCCGCAAAAAAGGG
AATAAGGGCGACACGGAAATGTTGAATACTCATACTCTTCCTTTTTCAATATTATTGAAGCATTTATCAGG
GTTATTGTCTCATGAGCGGATACATATTTGAATGTATTTAGAAAAATAAACAAATAGGGGTTCCGCGCAC
ATTTCCCCGAAAAGTGCCACCTGACGTCGACGGATCGGGAGATCTCCCGATCCCCTATGGTGCACTCTCA
GTACAATCTGCTCTGATGCCGCATAGTTAAGCCAGTATCTGCTCCCTGCTTGTGTGTTGGAGGTCGCTGA
GTAGTGCGCGAGCAAAATTTAAGCTACAACAAGGCAAGGCTTGACCGACAATTGCATGAAGAATCTGCT
TAGGGTTAGGCGTTTTGCGCTGCTTCGCGATGTACGGGCCAGATATACGCGTTGACATTGATTATTGACT
AGGCTTTTGCAAAAAGCTTTGCAAAGATGGATAAAGTTTTAAACAGAGAGGAATCTTTGCAGCTAATGG
ACCTTCTAGGTCTTGAAAGGAGTGCCTCGTGAGGCTCCGGTGCCCGTCAGTGGGCAGAGCGCACATCGC
CCACAGTCCCCGAGAAGTTGGGGGGAGGGGTCGGCAATTGAACCGGTGCCTAGAGAAGGTGGCGCGG
GGTAAACTGGGAAAGTGATGTCGTGTACTGGCTCCGCCTTTTTCCCGAGGGTGGGGGAGAACCGTATAT
AAGTGCAGTAGTCGCCGTGAACGTTCTTTTTCGCAACGGGTTTGCCGCCAGAACACAGGTAAGTGCCGT
GTGTGGTTCCCGCGGGCCTGGCCTCTTTACGGGTTATGGCCCTTGCGTGCCTTGAATTACTTCCACCTGG
CTGCAGTACGTGATTCTTGATCCCGAGCTTCGGGTTGGAAGTGGGTGGGAGAGTTCGAGGCCTTGCGCT
TAAGGAGCCCCTTCGCCTCGTGCTTGAGTTGAGGCCTGGCCTGGGCGCTGGGGCCGCCGCGTGCGAAT
CTGGTGGCACCTTCGCGCCTGTCTCGCTGCTTTCGATAAGTCTCTAGCCATTTAAAATTTTTGATGACCTG
CTGCGACGCTTTTTTTCTGGCAAGATAGTCTTGTAAATGCGGGCCAAGATCTGCACACTGGTATTTCGGT
TTTTGGGGCCGCGGGCGGCGACGGGGCCCGTGCGTCCCAGCGCACATGTTCGGCGAGGCGGGGCCTG
CGAGCGCGGCCACCGAGAATCGGACGGGGGTAGTCTCAAGCTGGCCGGCCTGCTCTGGTGCCTGGCCT
CGCGCCGCCGTGTATCGCCCCGCCCTGGGCGGCAAGGCTGGCCCGGTCGGCACCAGTTGCGTGAGCGG
AAAGATGGCCGCTTCCCGGCCCTGCTGCAGGGAGCTCAAAATGGAGGACGCGGCGCTCGGGAGAGCG
GGCGGGTGAGTCACCCACACAAAGGAAAAGGGCCTTTCCGTCCTCAGCCGTCGCTTCATGTGACTCCAC
GGAGTACCGGGCGCCGTCCAGGCACCTCGATTAGTTCTCGAGCTTTTGGAGTACGTCGTCTTTAGGTTG
GGGGGAGGGGTTTTATGCGATGGAGTTTCCCCACACTGAGTGGGTGGAGACTGAAGTTAGGCCAGCTT
GGCACTTGATGTAATTCTCCTTGGAATTTGCCCTTTTTGAGTTTGGATCTTGGTTCATTCTCAAGCCTCAG
ACAGTGGTTCAAAGTTTTTTTCTTCCATTTCAGGTGTCGTGAGGAATTAGCTTGGTACTAATACGACTCAC
TATAGGGAGACCCAAGCTGGCTAGGTAAGCTTGGTACCGAGCTCGGATCCACTAGTCCAGTGTGGTGG
AATTCTGCAGATATCAACAAGTTTGTACAAAAAAGCAGGCTCCGCCACCATGTCCGTCCTGACGCCGCTG
CTGCTGCGGGGCTTGACAGGCTCGGCCCGGCGGCTCCCAGTGCCGCGCGCCAAGATCCATTCGTTGATG
CACATGGTCAGCGAGCTGATCAAGGAAAACATGCACATGAAACTGTACATGGAGGGGACTGTGAACAA
TCACCATTTCAAATGCACCTCCGAGGGCGAAGGGAAGCCCTACGAGGGCACACAGACTATGAGGATCA
AGGCAGTGGAGGGAGGACCACTGCCATTCGCCTTTGACATTCTGGCTACCTCATTCATGTACGGCAGCA
AAACCTTCATCAATCACACTCAGGGGATTCCCGACTTCTTTAAGCAGTCTTTCCCTGAAGGCTTTACTTGG
GAGCGAGTGACCACATACGAGGATGGAGGCGTCCTGACCGCCACACAGGACACAAGTCTGCAGGATGG
CTGTCTGATCTATAACGTGAAGATTCGCGGGGTCAACTTTCCCAGTAATGGACCTGTGATGCAGAAGAA
AACCCTGGGATGGGAGGCTTCAACTGAAACCCTGTACCCAGCAGACGGAGGACTGGAGGGACGAGCA
GATATGGCTCTGAAACTGGTGGGCGGGGGACACCTGATCTGCAACCTGAAGACTACCTATCGGTCCAAG
AAACCTGCTAAGAATCTGAAAATGCCAGGCGTGTACTATGTGGACCGGAGACTGGAGAGAATTAAGGA

AGCAGATAAAGAGACCTACGTGGAGCAGCACGAAGTGGCTGTCGCACGATATTGTGACCTGCCTTCTAA
ACTGGGCCATCGGGGCGGGTCTATGGTGAGTAAGGGCGAGGAACTGTTCACAGGGGTGGTCCCAATCC
TGGTGGAACTGGACGGCGATGTCAATGGGCACAAGTTCAGCGTGTCCGGAGAGGGAGAAGGGGACGC
AACCTTTGGAGGCAGCGCCGACCTGGAAGATAATATGGAGACACTGAACGATAATCTGAAAGTGATCG
AGAAAGCCGACAACGCCGCTCAGGTCAAGGATGCTCTGACTAAAATGAGGGCAGCCGCTCTGGATGCA
CAGAAAGCCACCCCCCCTAAGCTGGAAGACAAATCACCTGATAGCCCAGAGATGAAGGACTTCCGCCAC
GGATTTGATATCCTGGTCGGCCAGATTGACGATGCTCTGAAGCTGGCAAATGAAGGCAAGGTGAAAGA
GGCACAGGCAGCCGCTGAGCAGCTGAAAACAACTAGGAACGCCTACCATCAGAAGTATCGCGGGGGAA
AGCTGACACTGAAATTCATCTGCACCACAGGCAAGCTGCCCGTGCCCTGGCCAACTCTGGTCACTACCCT
GGGATACGGCGTGCAGTGTTTTTCCCGCTATCCAGACCACATGAAGCAGCATGATTTCTTTAAATCTGCC
ATGCCCGAAGGCTACGTGCAGGAGAGAACCATCTTCTTTAAGGACGATGGAAACTATAAAACAAGGGC
TGAAGTGAAGTTCGAGGGAGACACTCTGGTCAACCGCATCGAACTGAAGGGCATTGACTTTAAAGAGG
ATGGAAATATTCTGGGCCACAAGCTGGAATACAACTATAATAGCCATAACGTGTACATCATGGCCGATA
AGCAGAAAAACGGCATTAAGGTCAATTTCAAAATCCGGCACAATATTGAGGACGGGAGCGTGCAGCTG
GCCGATCATTACCAGCAGAACACCCCAATCGGGGACGGACCAGTGCTGCTGCCCGATAATCACTATCTG
TCCACACAGTCTGCCCTGAGTAAGGACCCTAACGAAAAAAGAGATCACATGGTGCTGCTGGAGTTTGTC
ACCGCAGCCGGGATTACACTGGGAATGGACGAGCTGTACAAGTGAGACCCAGCTTT

2. Supporting Figures

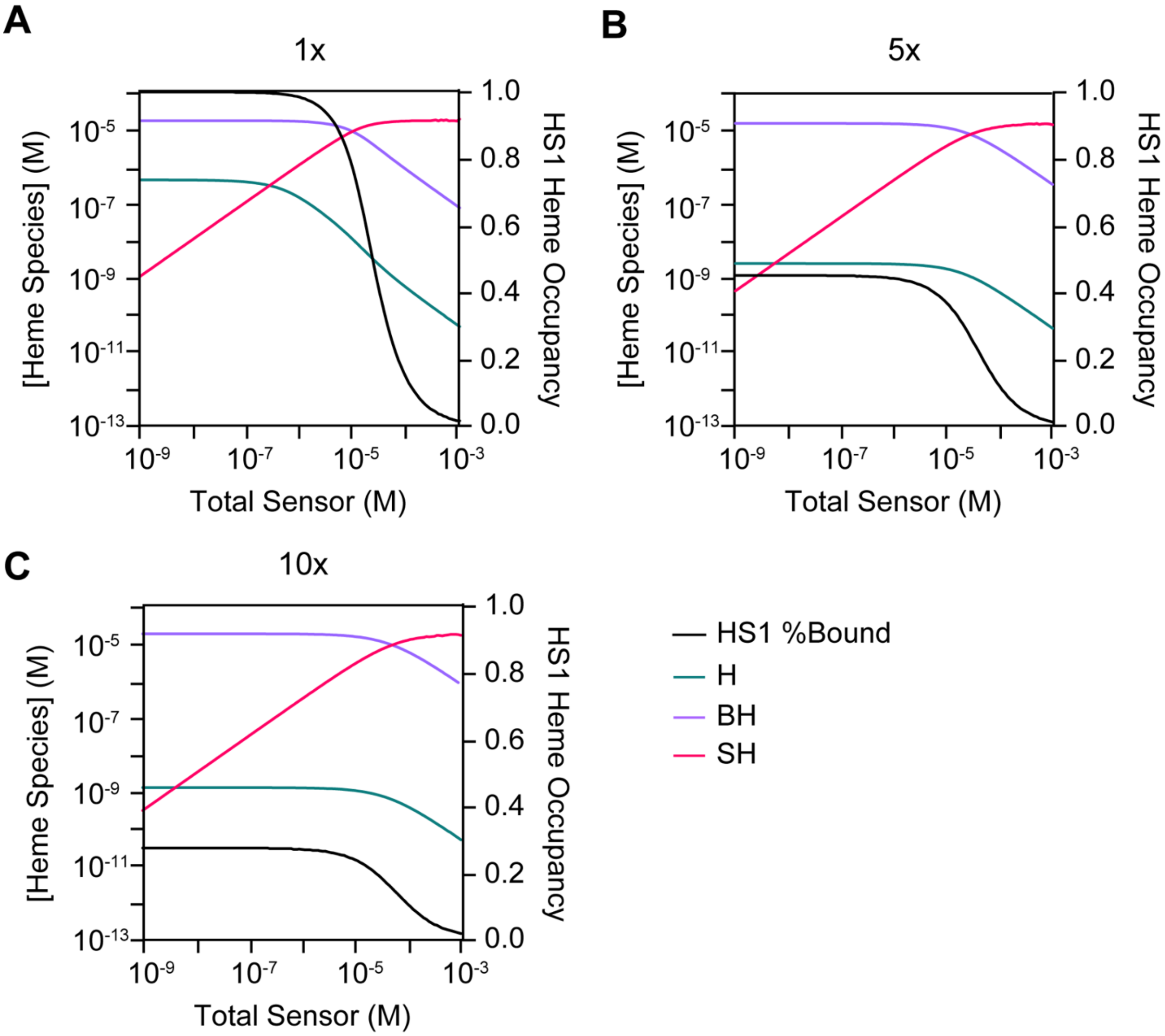

Figure S1. The relationship between sensor heme occupancy (black, right y-axis) and concentration of heme species (left y-axis) is simulated as a function of heme sensor expression for three different heme buffer concentrations, 15 μM (1x; **A**), 75 μM (5x; **B**), and 150 μM (10x; **C**) using the mass balance relationships below in ChemEQL (v. 3.2.1)

$[H]_{Total} = [H] + [B\text{-}H] + [S\text{-}H]$

$[S]_{Total} = [S] + [S\text{-}H]$

$[B]_{Total} = [B] + [B\text{-}H]$

H is unbound free heme, S is apo heme sensor, B represents a competing apo hemoprotein that can buffer heme, S-H is the heme bound state of sensor, and B-H is the heme bound state of the hypothetical hemoprotein that constitutes the heme buffering system. The heme sensor, S-H, K_D is assumed to be 3 nM. Total heme is assumed to be 15 μM, which is similar in magnitude to the 10-30 μM heme levels measured in HEK293 cells (this study). For reference, typical HS1 expression levels in cells is ~10-100 nM. However, the heme buffer concentration $[B]_{Total}$ and the heme affinities of the buffer (assumed to have a K_D = 10 nM), are unknown and likely constitute a heterogenous mix of different hemoprotein ligands. Simulations were conducted using ChemEQL (v. 3.2.1) (1,2).

S2

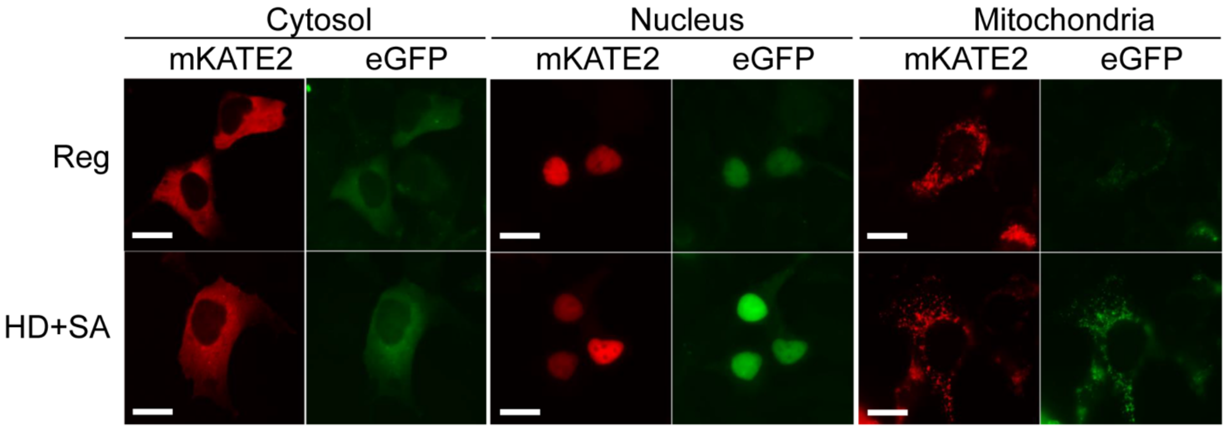

Figure S2. Localization of targeted hHS1 reporters. HEK293 cells were transfected with pEF5/FRT/V5-DEST-hHS1 constructs targeted to the cytosol, nucleus and mitochondria. Images were taken 42 hours post-transfection using a Leica DM IRE2 fluorescent microscope under a 63x oil immersion objective. Scale bar = 10 µm.

S3

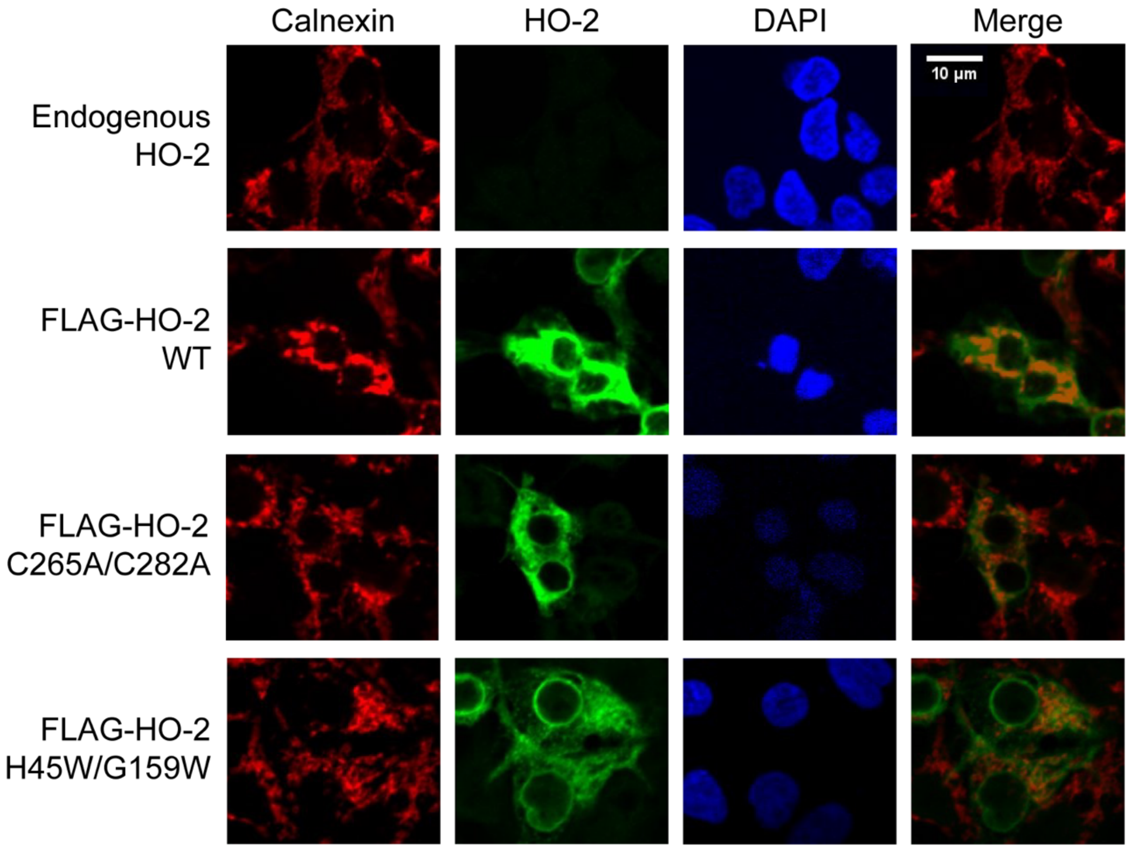

Figure S3. HO2 and its variants localize to the ER. HEK293 cells cultured on coverslips were transfected with transfection reagent only (Endogenous HO2) or with corresponding expression vector for FLAG-tagged HO2 and its variants as indicated. Endogenous HO2 was stained with anti-HO2 antibody, FLAG-tagged HO2 was probed with anti-FLAG antibody. Calnexin was used as a marker for ER and DAPI was used to show the position of nuclei. “Merge” is the merged images of Calnexin channel and HO2 channel. All images are in the same scale as indicated with the 10 µm scale bar.

S4

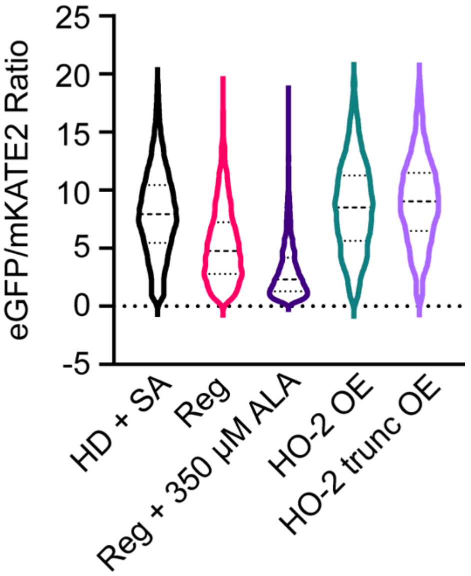

Figure S4. Deletion of the membrane spanning region of HO-2, 288-316 (HO-2 trunc), which tethers HO-2 to the ER membrane, does not affect the ability of HO-2 to deplete labile heme when overexpressed (OE). Cells were analyzed by flow cytometry as described in **Experimental Procedures** and cultured to be heme depleted (HD+SA), heme replete (Reg), or have excess heme (Reg + 350 µM ALA).

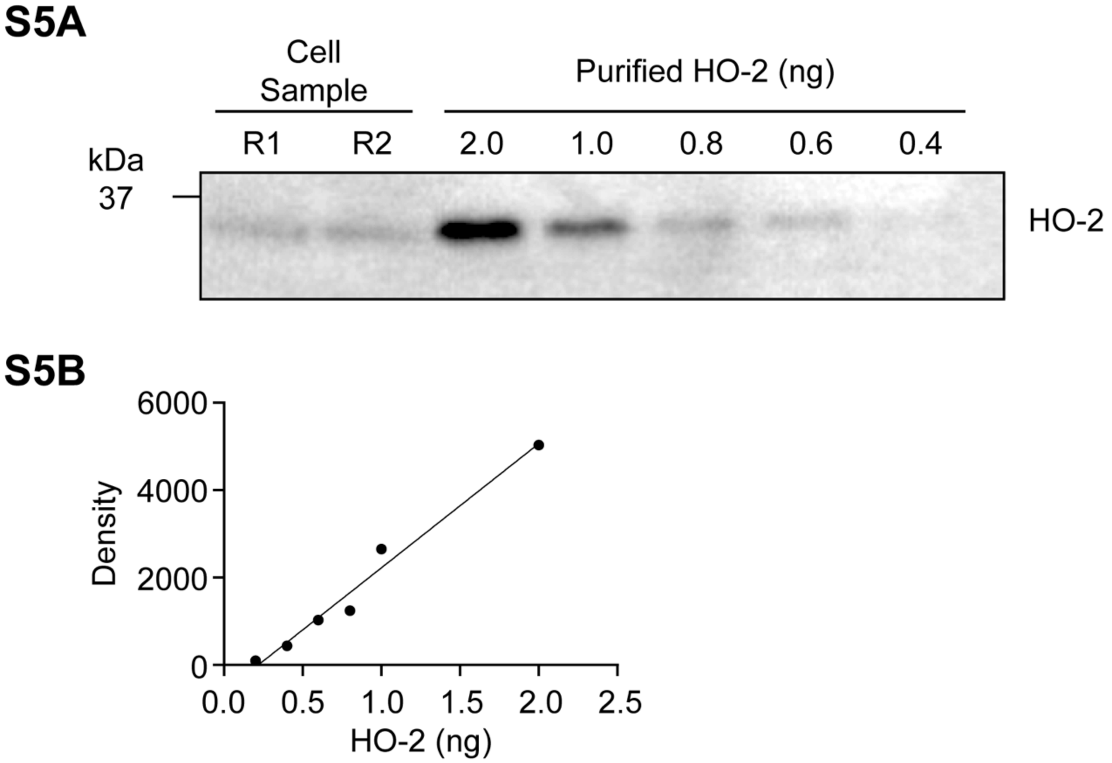

Figure S5. Quantitative immunoblotting to determine the concentration of endogenous HO-2 expression in HEK293 cells. Analysis reveals that HO-2 is expressed at a concentration of ~10 nM, assuming a HEK293 cell volume of 1.2 pL.

S6

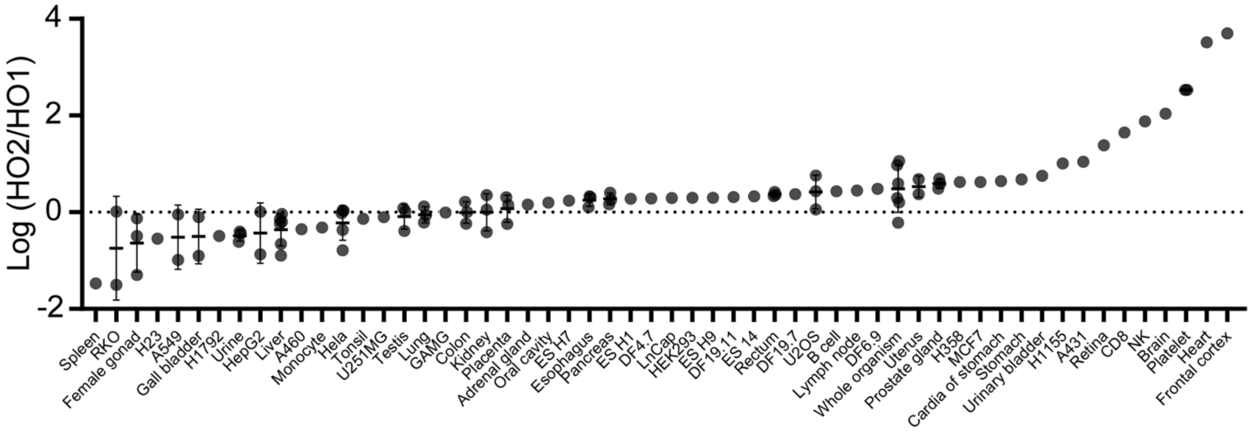

Figure S6. Relative HO-2 and HO-1 protein expression across various cell types and tissues as assessed by quantitative proteomics (3,4).

S7

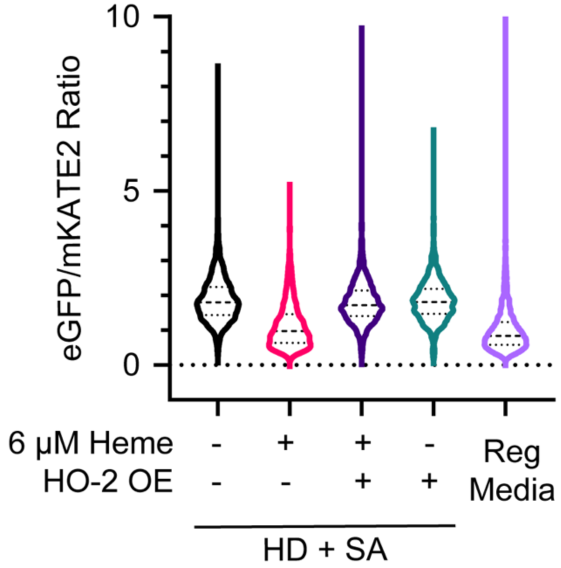

Figure S7. Overexpression of HO-2 is able to deplete mitochondrial labile heme if heme is supplied exogenously. Cells were analyzed by flow cytometry as described in **Experimental Procedures** and cultured to be heme depleted (HD+SA), heme replete (Reg), or supplemented with the indicated concentration of heme. This is in contrast to endogenously synthesized labile heme in the mitochondria, which is unaffected by HO-2 overexpression (**Figure 4**).

S8a

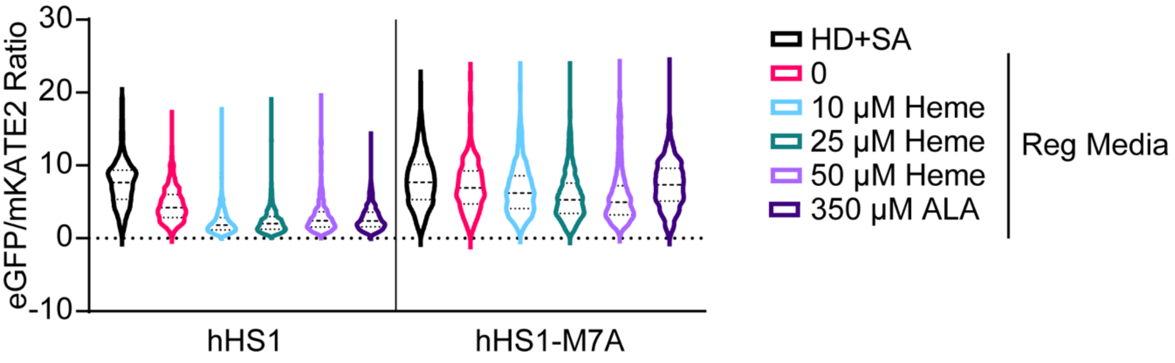

S8b

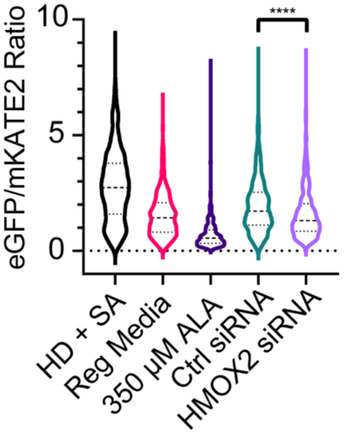

S8c

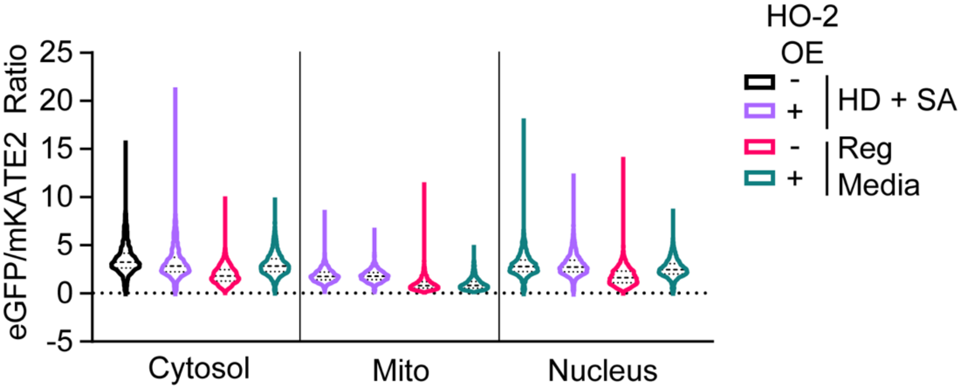

Figure S8. Representative flow cytometry histograms, as depicted using violin plots, of HS1 eGFP/mKATE2 fluorescence ratios in HEK293 cells expressing the indicated sensors. (**a**) Representative violin plots of heme sensor eGFP/mKATE2 fluorescence ratio distributions from single cell analysis of HEK293 cultures are shown for cells grown in HD + SA media or in regular media supplemented with the indicated concentrations of hemin chloride or 5-aminolevulinic

acid (ALA) for 24 hours. See **Figure 2b**. (**b**) Representative violin plots of heme sensor eGFP/mKATE2 fluorescence ratio distributions from single cell analysis of HEK293 cultures are shown for HEK293 grown in HD + SA media, regular media, or regular media supplemented with 350 µM ALA or control or targeted siRNA against HMOX2. See **Figure 3b**. (**c**) Representative violin plots of heme sensor eGFP/mKATE2 fluorescence ratio distributions from single cell analysis of HEK293 cultures are shown for HEK293 cells expressing cytosolic, nuclear or mitochondrial (mito)-targeted HS1 in untranfected (-) or HO-2 overexpressing (OE) (+) HEK293 cells grown in HD + SA or regular media.

S9

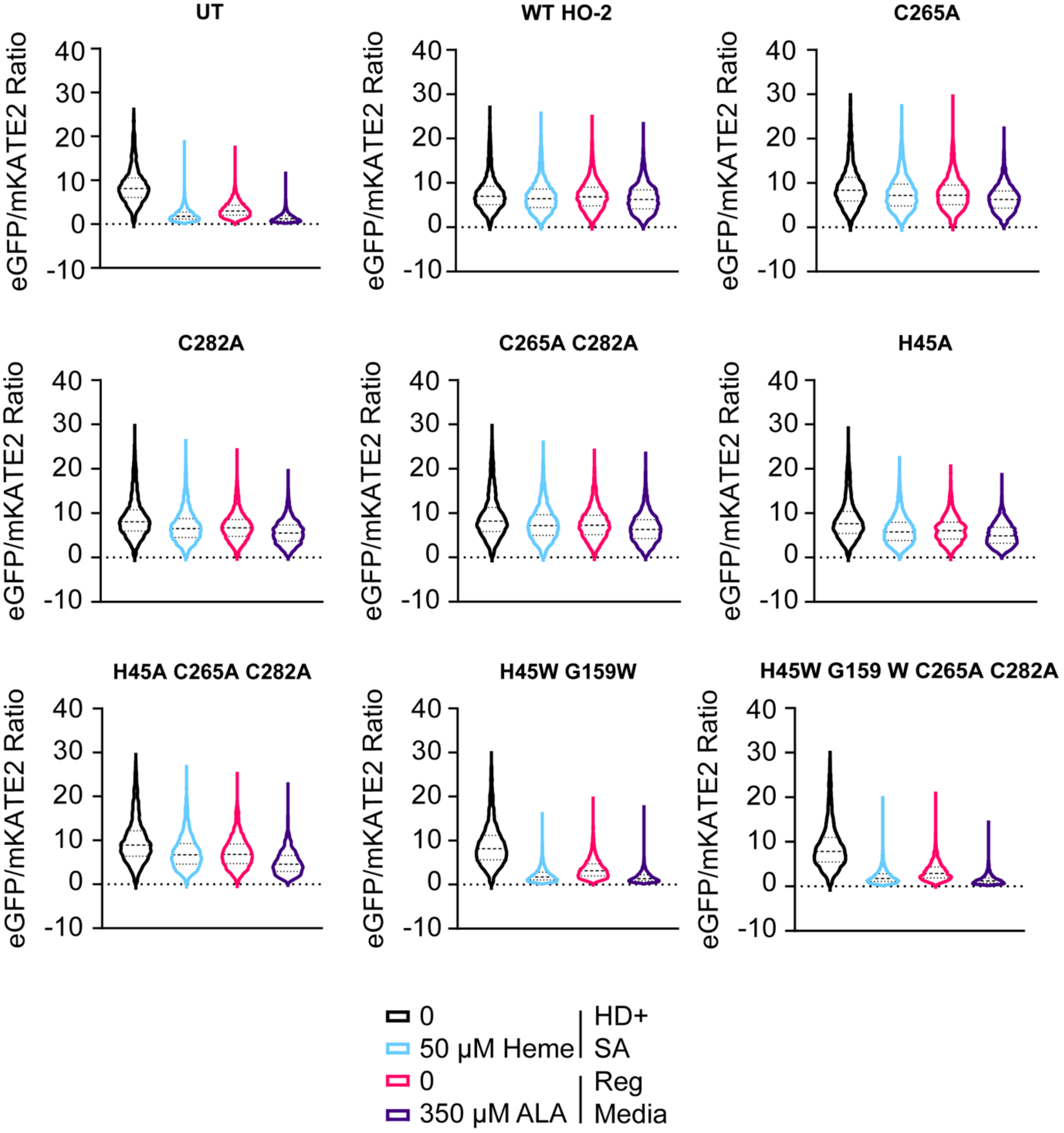

Figure S9. Representative flow cytometry histograms, as depicted using violin plots, of HS1 eGFP/mKATE2 fluorescence ratios in HEK293 cells expressing the indicated HO-2 variants grown in HD + SA media or in regular media supplemented with the indicated concentrations of hemin chloride or 5-aminolevulinic acid (ALA) for 24 hours. See **Figure 5b**.

Supporting References

1. Westall, J. C., Zachary, J. L., and Morel, F. M. M. (1976) Mineql - General Algorithm for Computation of Chemical-Equilibrium in Aqueous Systems. *Abstracts of Papers of the American Chemical Society* **172**, 8-8
2. Müller, B. (1996) ChemEQL V. 2.0. A program to calculate chemical speciation and chemical equilibria.
3. Wang, M., Herrmann, C. J., Simonovic, M., Szklarczyk, D., and von Mering, C. (2015) Version 4.0 of PaxDb: Protein abundance data, integrated across model organisms, tissues, and cell-lines. *Proteomics* **15**, 3163-3168
4. Geiger, T., Wehner, A., Schaab, C., Cox, J., and Mann, M. (2012) Comparative proteomic analysis of eleven common cell lines reveals ubiquitous but varying expression of most proteins. *Mol Cell Proteomics* **11**, M111 014050
